# Efficient log *P* determination by automated, spatially encoded ^19^F NMR spectroscopy

**DOI:** 10.1039/d5md00678c

**Published:** 2026-01-12

**Authors:** Susanna H. Wood, Fiona Gordon, Robin S. Stein, Mark J. Howard, John A. Parkinson

**Affiliations:** a Department of Pure and Applied Chemistry, University of Strathclyde Thomas Graham Building, 295 Cathedral Street Glasgow G1 1XL UK susanna.h.wood@strath.ac.uk; b Bruker (UK) Ltd. Welland House, Westwood Business Park, Longwood Close Coventry CV4 8HZ UK

## Abstract

We report a straightforward method for direct determination of log *P* by slice-selective ^19^F NMR spectroscopy utilising a single NMR sample. Our technique uses a partitioned NMR sample of *n*-octanol/water containing the compound of interest, a modern NMR spectrometer equipped with ^19^F-observation and *Z*-axis pulsed-field gradient capabilities in addition to automation programs to measure solute distribution between solvent layers. The approach is validated using a range of fluorinated compounds with known experimental log *P* values. Details of our experimental method development and implementation processes are reported in supporting data and the benefits of using this approach for experimental log *P* determination are highlighted.

## Abstract 2

A method for measuring log *P* of fluorinated molecules by ^19^F NMR under full instrument automation. Placing an advanced NMR technique in the hands of synthetic chemists for simple, efficient data acquisition.

## Graphical abstract

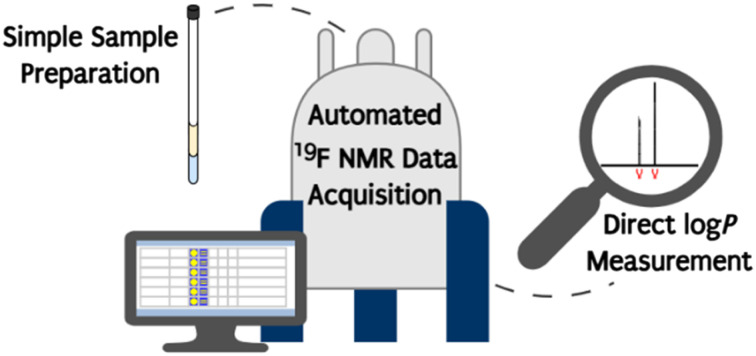

## Introduction

Organofluorine compounds are prevalent in the field of medicinal chemistry, with fluorination often being used to modulate the properties of small molecules, including their partitioning characteristics.^1–3^ The logarithm of the partition coefficient between *n*-octanol and water (log *P* or log *K*_ow_) is used extensively to indicate how molecules distribute between water and lipid (fat) environments. In bioactive molecule development, log *P* is used as an indicator of a molecule's ability to permeate through the blood–brain barrier or to diffuse across cell membranes and is therefore an important physicochemical property for drug discovery campaigns.^4^ Historically, log *P* is measured directly through the partitioning of a compound between *n*-octanol and water (the ‘shake flask’ method) where the compound is stirred or shaken in a 1 : 1 mixture of *n*-octanol and water. The phases are allowed to separate; each phase is sampled and the concentration of analyte in each phase is then quantified.^5^ This method has many drawbacks, which prevent synthetic and medicinal chemists from routinely carrying out these measurements upon synthesis of a molecule. For example, there are difficulties in sampling the aqueous phase without also including some *n*-octanol phase. Such cross-contamination could lead to inaccuracies in the measurement. Additionally, the analytical methods commonly employed in log *P* measurements can be problematic. For instance, UV/vis spectroscopy relies on molecules containing a chromophore, high performance liquid chromatography (HPLC) and gas chromatography require calibration curves to be prepared. An alternative to the traditional ‘shake flask’ method for measuring log *P* is the HPLC-based chromLog *P* approach.^6^ This has gathered widespread adoption due to the commonplace availability of HPLC analysis in modern synthetic laboratories. In this method, log *P* is estimated from the correlation between known values of log *P* determined by the ‘shake flask’ method and the retention time on a HPLC column. Whilst the HPLC approach is operationally simpler than traditional ‘shake flask’ protocols, it still requires the time-consuming generation of calibration curves and then delivers chromLog *P* values within ±0.5.^7^ When considered in the context of a logarithmic scale, such a margin has the potential for large degrees of error. In addition, the technique is unsuitable for molecules which are strong acids or bases or exist as transition metal complexes. Despite its widespread utility, the experimental challenges associated with the measurement of log *P* often restrict reported values to those computationally derived, rather than experimentally determined. Depending on the computational protocol employed, the degree of accuracy of calculated log *P* values can vary.^8–13^ Considering the widespread use of organofluorine compounds in medicinal chemistry^14–18^ routine, accurate quantification of log *P* for fluorinated molecules using robust and familiar experimental techniques could prove invaluable.

Significant progress towards this goal has been made in recent years through the development of protocols that exploit NMR spectroscopy as the analytical technique of choice for quantifying solute partitioning between *n*-octanol and water phases.^19–21^ Work by Linclau *et al.*^22^ exploited the absence of background solvent signals in ^19^F NMR to allow the measurement of log *P* for a range of non-UV active fluorinated molecules. This technique has since been expanded to allow log *P* measurements to be made on non-fluorinated molecules by using ^31^P^23^ and ^1^H NMR spectroscopy instead.^24^ This method is advantageous and attractive to the synthetic chemist due to its simplicity through acquisition of the relevant NMR spectra. However, the technique does have some significant drawbacks. The set-up and sample preparation for the experiment is time consuming: the experimentalist must set up a traditional ‘shake flask’ experiment, in which the compound of interest, alongside a reference compound of known log *P*, are introduced into a vessel along with *n*-octanol and water; the phases must then be mixed and allowed to separate; two samples must then be taken, one from each phase, taking care to avoid contaminating either phase with the other; each sample is then separately analysed by ^19^F, ^31^P or ^1^H NMR as appropriate with the log *P* value then being calculated from the relative integrals of the compound of interest and the reference. The authors report that the reference compound must have a log *P* value similar enough to the compound of interest to yield an integral ratio between 0.1 and 10. When combined with the need for the NMR signals to appear relatively near each other in the spectrum to facilitate improved signal-to-noise from a narrower spectral width, these combined conditions pose a challenge for the log *P* determination of fluorinated molecules due to the broad spectral range associated with ^19^F NMR and the need to find a molecule with a resonance in a similar region which has a measured log *P* within the given range. Methods using ^31^P nuclei are limited by the relatively low abundance of compounds containing phosphorus atoms in a synthetic and medicinal chemistry context, in addition to the comparably lower sensitivity of the nucleus as well as issues with selecting a known reference. The ^1^H NMR methods are limited by the background solvent signals associated with *n*-octanol and water as well as selection of a suitable reference compound.

Taking into account all the restrictions associated with effective sampling of *n*-octanol and water phases under ‘shake flask’ conditions, other NMR approaches have been developed, including the use of band selective^25^ and slice selective^26–28^^1^H NMR methods. Such methods are usually sophisticated in their setup, calibration and execution and may be considered to sit beyond the domain of ‘NMR users’, including synthetic chemists as opposed to NMR specialists. Recent work^26^ has simplified much of this process by providing an automated ^1^H NMR protocol for the direct measurement of slice selective spectra of partitioned samples. In this procedure, samples of analyte were partitioned between *n*-octanol and water along with either an internal or external reference compound. Slice selective ^1^H NMR spectra were then acquired and the log *P* value was then calculated from the integral ratios in the analyte spectra from both the *n*-octanol and the water phases in a similar fashion used by the ^19^F, ^31^P and ^1^H examples discussed above. The only difference in the slice selective approach is that the two sets of spectra are generated from a single sample. This method greatly lessens the experimental sample preparation workload. However, it does not resolve the issues surrounding spectral crowding in ^1^H NMR data (limiting the measurement's applicability mostly to molecules with signals in the aromatic region) or eliminate the need for the selection of a suitable reference compound. In addition, only a limited range (−1.35 to 1.66) of log *P* values could be demonstrated.

The approach adopted in this work uses ^19^F NMR spectroscopy and demonstrates access to a wider range of log *P*, including the measurement of a 0.024 M sample of trifluorotoluene (log *P* = 3.01) with an acquisition time of around half an hour. Once incorporated into a typical NMR spectrometer automation protocol, the procedure is made readily accessible for typical NMR users by isolating the complexities of slice selective NMR experiment set-up to background processes. In the absence of a standard reference, slice selective ^19^F NMR data are acquired on solute molecules containing at least one fluorine atom and partitioned between two immiscible solvent layers within the same NMR sample. This allows the acquisition of spectra from both layers, on a single sample, with no background solvent signal, and with excellent sensitivity. The approach is attractive in its simplicity, efficiency and delivery: an analyte is admitted to a 5 mm NMR tube; equal volumes of water and *n*-octanol are added; the tube is shaken vigorously; the layers are left to separate; ^19^F NMR spectra are acquired on each layer within the same sample under full automation; data are extracted and integrated from which log *P* is determined. Following initial setup of the experiment on the NMR spectrometer, the method can be implemented under full automation by selection of the experiment from a drop-down menu in the NMR automation programme, therefore, requiring minimal input from the experimentalist. Here we report the development of this ^19^F slice-selective NMR procedure. We demonstrate an operationally simple log *P* determination method operating under full automation and include pulse programs and automation (AU) programs suitable for use on modern Bruker NMR spectrometers together with instructions for implementing the procedure. Its utility in generating a broad range of experimental log *P* values under efficient, medium to fast throughput for a selection of fluorinated molecules is illustrated.

## Results and discussion

Originally, a variation on the method described by Linclau *et al.*^22^ was envisioned, where the analyte is measured in the presence of a known reference compound, the variation being that the *n*-octanol and water phase measurements would result from the same sample. To this end, trifluoroethanol and hexafluoroisopropanol were added to an NMR tube, followed by 200 μL *n*-octanol and 200 μL water. The mixture was shaken vigorously and the phases allowed to separate. A capillary containing D_2_O was introduced into the NMR tube and a slice selective measurement of ^19^F NMR spectra from each phase was carried out. These initial measurements used an approach taken from the work of Mitrev^27^ whereby a spin-echo pulse sequence was adapted using selective-90° and selective-180° pulses over pulsed-field gradients, which provides the spatial encoding element (See Fig. S4a in the SI). Initial ^19^F NMR data were acquired without ^1^H decoupling at a magnetic field strength of 9.4 T using a room-temperature BBFO-z probe (later replaced during this work with BBFO-z SmartProbe [iProbe]). Slice thickness, Δ*z*, is proportional to pulse bandwidth, *BW*, and inversely proportional to gradient strength, *G*, and *γ*, the gyromagnetic ratio, (eqn (1)). By setting the ^19^F pulse bandwidth to 6 kHz for both shaped pulses (90° G4 cascade = 1303.33 μs, 180° RSnob = 388.67 μs) each applied during square z-pulsed field gradients of 16% (equivalent to 8.8 G cm^−1^), a slice thickness of 1.7 mm was achieved. The physical position of the slices (see eqn (2)) was set by offsetting each pulse using the Bruker spoffs parameter. Offsetting both pulses initially by −21.5 kHz led to slice selection from the lower, aqueous phase at a distance of 6.1 mm from the solvent interface. By contrast, offsetting both pulses by +21.5 kHz led to slice selection from the upper, octanol phase 6.1 mm from the solvent interface.1
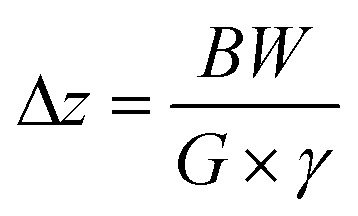


In this way, two spectra were acquired, each using 16 transients, 2 dummy transients and a relaxation delay of 30 s between each transient. The approach produced one spectrum associated with the *n*-octanol phase and one spectrum from the aqueous phase, allowing calculation of the log *P via* the established method (in a similar fashion to Ben-Tal *et al.*,^26^ only using ^19^F rather than ^1^H as the observe nucleus). When processing the data, it was apparent that there was a significant chemical shift change for the ^19^F signals from each component when analysing the spectra from the *n*-octanol phase relative to the chemical shift in the aqueous phase. This difference allowed the spectra to be summed, when it became apparent that log *P* could be derived directly from the integral ratios of the ^19^F NMR signals from each species in the aqueous and *n*-octanol phases independently, without requiring the use of the signals from the other ‘reference’ species. This led to the development of the reference-free approach. The minimum relaxation delay required to produce accurate data was found to be 30 s; any reduction in this delay results in erosion of the technique's accuracy. The duration of the relaxation delay was kept the same for the measurement of each phase in the method. While ^19^F T_2_ may typically be short, ^19^F T_1_ for small molecules remains long. Setting a relaxation delay equal to or greater than 5 × T_1_ for automated data acquisitions ensures that integrals are reliable, reflecting full recovery equilibrium spin magnetization. The functionality was tested under manual control and found to provide suitable log *P* measurements (see SI).

Given the widespread utility of log *P* in understanding the behaviour of organic molecules, we anticipated that this method of log *P* measurement would be of interest to synthetic and medicinal chemists due to the ease of sample preparation. However, use of the advanced NMR technique described (requiring pulse calibrations, gradient and offset setting and related adjustments) alongside operation of the spectrometer under manual control in the context of a busy, high throughput, generally automated setting, presented a significant barrier to general use of the technique, as a high degree of expertise in NMR spectroscopy would be required to utilise the method routinely in this form. In order to place this technique in the hands of synthetic experimentalists who are comfortable with running an array of NMR experiments by controlling the spectrometer under automated programmes (using Bruker's IconNMR in this case) we set about developing the described technique into an automated process for running the NMR experiment. To obtain the highest quality data, it was beneficial for the signal of interest to be centred in the NMR spectrum (close to the frequency offset, o1p), and for the spectral width to be relatively narrow (10 ppm) to ensure adequate to good signal digitization.

To automate this smoothly, an initial scout scan on the sample was incorporated into the procedure to determine the frequencies of the largest peaks, thereby allowing the frequency offset, o1p, to be set automatically and be subsequently used in the slice selective ^19^F NMR experiments automatically run to acquire the necessary data. Once acquired, the spectra from the *n*-octanol and the water phases were separately stored and also automatically summed, resulting in a dataset consisting of four NMR spectra: a pseudo 2D ^19^F NMR spectrum, the *n*-octanol phase ^19^F NMR spectrum, the water phase ^19^F NMR spectrum and the summed ^19^F NMR spectrum. Assuming the *n*-octanol phase ^19^F NMR signal and the aqueous phase ^19^F NMR signal are sufficiently displaced from one another, the two signals can be directly integrated and the log of the integral ratio will then be equal to the log *P* value. Should the chemical shift displacement of the signals be insufficient to fully resolve each signal in the automatically summed spectrum, they can be displaced manually following inspection of the data and summed to provide adequate spectral resolution. For samples with log *P* close to zero, where the molecule is well distributed between the two phases, this can result in the signal being insufficiently strong during the scout scan, resulting in the o1p being set to an incorrect position and no signals appearing in the acquired spectra. This scenario was encountered when attempting to measure the log *P* of a 0.18 M sample of 1-fluoroethanol (log *P* −0.68). To rectify this, the number of scans was increased to 16, resulting in correct location of the signals and log *P* measurement of −0.73.

To probe how generally applicable the procedure of slice-selective ^19^F NMR followed by spectral summation was for the measurement of log *P*, a series of compounds were measured by this method. To prepare the samples, a small amount (0.013–0.210 mmol, see SI for details) of analyte was introduced into the NMR tube, followed by the addition of equal volumes of *n*-octanol and water measured by syringe or micropipette. The volume of *n*-octanol and water added to the sample was dependent on the intended length of the NMR acquisition – for short acquisitions (typically less than 1 hour duration), 270 μL of *n*-octanol and water are added, for acquisitions longer than 1 hour 200 μL of each solvent were added. The tube was capped and vigorously shaken to solubilise the analyte and to facilitate partitioning. The phases are then allowed to separate. For some samples, to access full separation of the phases, centrifugation of the NMR tube was necessary, for instance using a hand-cranked centrifuge. For long acquisitions (200 μL each of *n*-octanol and water used), a capillary containing a small volume of D_2_O was then introduced into the NMR tube to provide a ^2^H lock signal for the NMR instrument. The simplicity and efficiency of the operation was enhanced further by eliminating the need to incorporate a ^2^H lock capillary for short duration experiments (see SI for commentary). Accuracy of log *P* determination is retained in the absence of sample ^2^H lock, but for long runs, inclusion of a ^2^H capillary is recommended to maintain satisfactory NMR lineshape. The NMR tube was placed into a spinner and the sample position was manually adjusted using a NMR sample depth gauge to ensure that the interface between the two phases was located correctly and coincides with the centre of the radio-frequency (rf) coil of the NMR probe. The NMR sample was loaded into the spectrometer from an automation carousel.

**Fig. 1:**
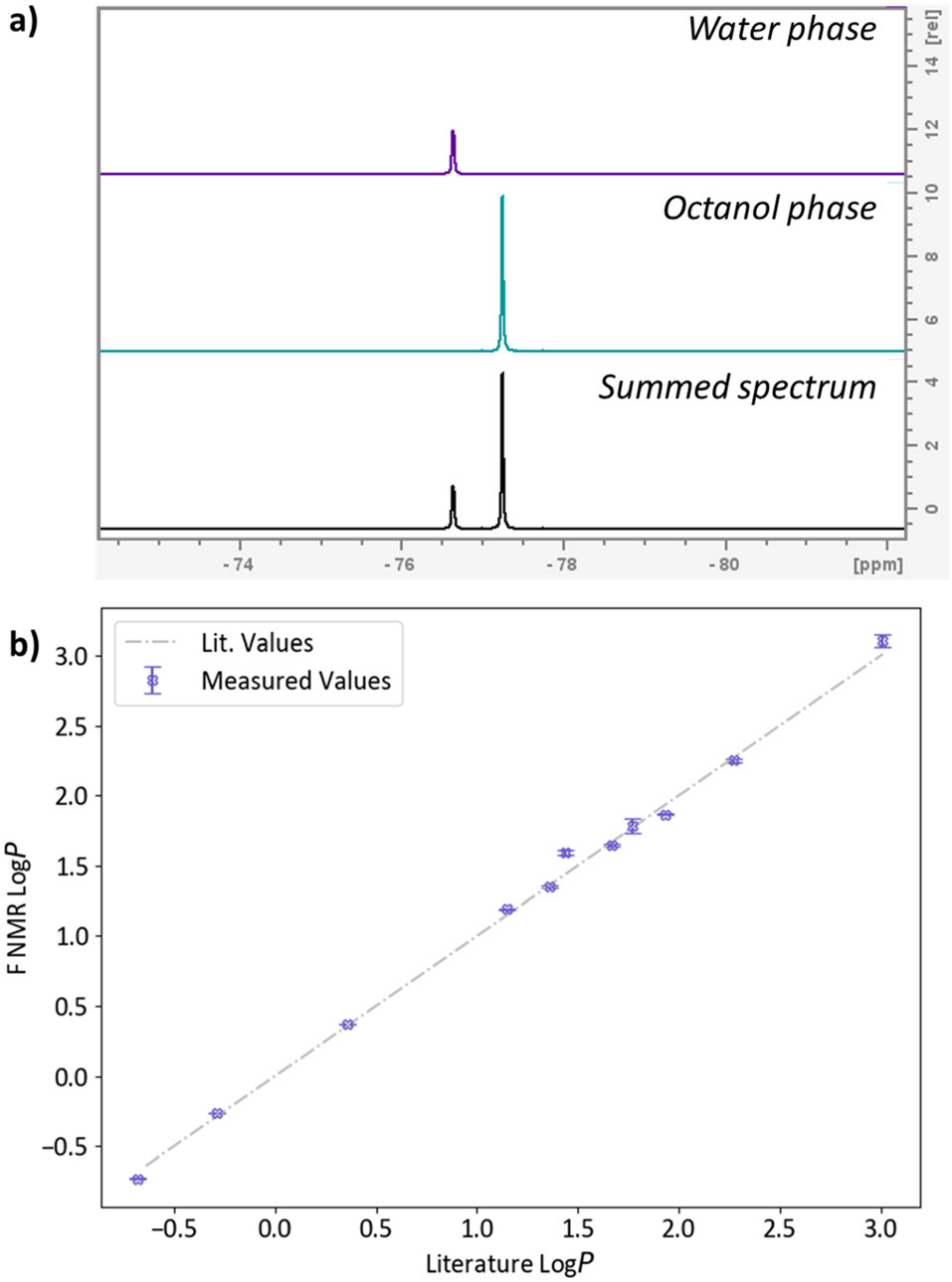
a) Partition spectra of trifluoroethanol b) literature values^22,29–32^ of log *P vs.*^19^F NMR measured at 9.4 T with BBFO-z probe.

Prior to slice-selective ^19^F NMR data acquisition of (a series of) partition samples, the NMR spectrum of a fluorinated species may be acquired (in this study trifluoroethanol in D_2_O was used), a step used to optimise and check magnetic field homogeneity and set the room temperature shims for the subsequent (series of) partition sample(s). ^19^F or if required ^19^F-{^1^H} spatially encoded NMR spectra of each phase of the partition sample are then acquired and processed. The final versions of the slice-selective ^19^F and ^19^F-{^1^H} pulse sequences follow the regime d1–90°-τ-(sel180°/grad)-τ-acquire, where d1 is the relaxation delay, 90° represents a hard, non-selective preparation pulse, sel180°/grad defines the spatial encoding element and τ is the echo delay. The output of the automated procedure comprises four files: a pseudo 2D spectrum containing the raw NMR data, two separate spectra extracted from the pseudo 2D spectrum, one from each of the *n*-octanol and water phases and the summation spectrum of the two separately extracted spectra (Fig. 1a), the latter being generally used to calculate log *P*. The partition coefficient is calculated directly from the integral ratio between the two signals in the summation spectrum. This procedure allowed log *P* to be determined for several compounds in excellent agreement with their previously measured and published values (Fig. 1b), with a maximum deviation from the literature value of 0.15 log units.

Slice selective NMR experiments are much less sensitive than the equivalent conventional NMR experiment due to the much smaller portion of sample being analysed in a given slice. This led to concerns regarding whether low signal-to-noise would affect the accuracy of the results.

To investigate this, the data reported in this study were processed according to a consistent method. Each signal was integrated across a 1 ppm chemical shift range, with the signal centred in each case, and the log *P* calculated from the integral ratio. The error from the measurement was propagated from the signal-to-noise ratio. Similar to the findings of Linclau *et al.*,^22^ low levels of signal-to-noise were tolerated for the integration of the smallest peak, yielding tolerable errors (up to 0.07 log units, see SI for details). The signal-to-noise associated with the smallest peak can be improved by integrating the spectra associated with the *n*-octanol phase and water phases without summing them and utilising the absolute integral value, with each spectrum scaled by the same factor. When compared with calculating the log *P* based on the integrals from a summed spectrum, the deviation from the value calculated using the individual spectra was observed to be a maximum of 0.04 log units in this study. Separate integration is recommended for spectra where one of the peaks is very small and signal-to-noise in the summed spectrum as dropped below 10 : 1. Despite this, in this study, satisfactory results were obtained by integrating the spectra separately with signal-to-noise as low as 8.01 : 1, much lower than would typically be tolerated for quantitative concentration measurements.^32^ This indicates that reliable log *P* measurements can be obtained through this method without the requirement for excessively high signal-to-noise. For enhanced confidence in the measurement, it is recommended that signal-to-noise of at least 10 : 1 is obtained for the smallest peak. To improve the signal-to-noise, an increasing number of transients can be acquired. However, this significantly extends the duration of the experiment. For example, the measurement of a 0.34 M sample of trifluorotoluene (log *P* = 3.01, where almost all of the analyte is present in the *n*-octanol phase, resulting in a concentration of around 0.68 M in the *n*-octanol phase), required 96 transients to observe the signal associated with the aqueous phase when using a 400 MHz instrument equipped with a BBFO-z probe, resulting in an experiment time of over an hour and a half, producing a log *P* measurement of 3.10 ± 0.04 with a signal-to-noise of the smallest peak of 10.46 : 1 (0.09 log units higher than the reported value). It should be noted that accurate measurement of log *P* of more extreme values at higher concentrations will be subject to their respective aqueous or *n*-octanol solubility in addition to the effect of any intermolecular interaction. In contrast, a sample of trifluorotoluene of much higher dilution (0.024 M) could be analysed by this method in 35 minutes using a 600 MHz instrument equipped with a H/F-C-N-z helium cryoprobe. Pleasingly, log *P* of 3.04 ± 0.04 could be measured on this instrument with only 32 transients per FID within error of the published value, with a signal-to-noise of 11.21 for the smallest peak.

**Fig. 2:**
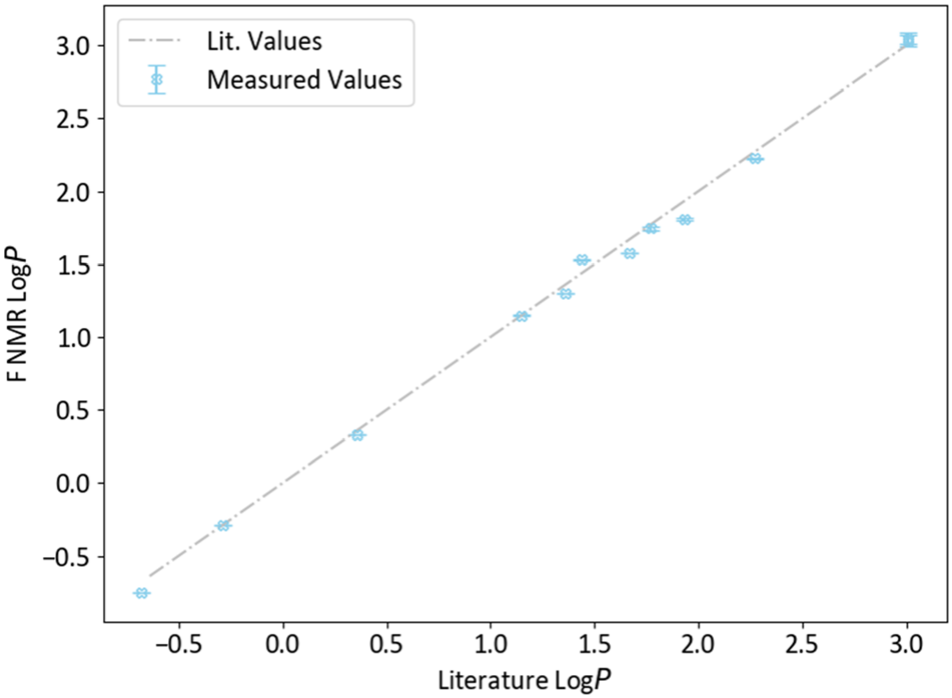
Literature values^22,29–31^ of log *P vs.*^19^F NMR measured at 14.1 T with helium cryoprobe probe.

To provide a direct comparison in performance with the 400 MHz instrument, each of the samples were run on the 600 MHz instrument equipped with a H/F-C-N helium cryoprobe (Fig. 2). For each of the samples, significant reduction in the number of transients was observed with only two transients required to acquire adequate spectra, with deviation in the log *P* of a maximum 0.07 log units when compared to measurements with a higher number of transients acquired on the 400 MHz instrument (see SI for details).

**Fig. 3:**
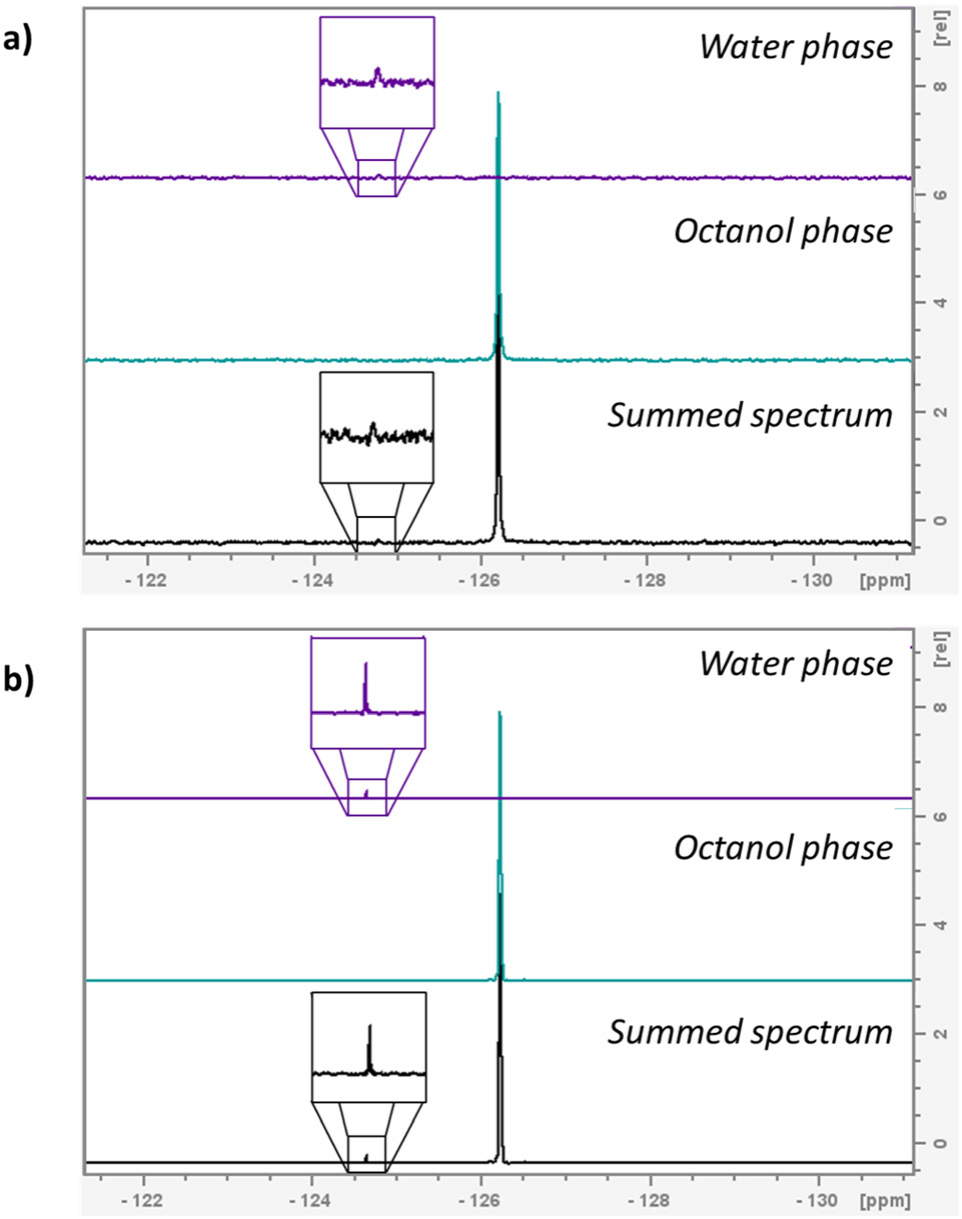
a) Partition spectra of 4-fluorophenol measured on 400 MHz spectrometer b) partition spectra of the same sample of 4-fluorophenol measured on 600 MHz instrument with he cryoprobe.

To further exemplify the enhancement in sensitivity when using the 600 MHz instrument equipped with the H/F–C–N helium cryoprobe, the spectra obtained from a 0.05 M sample of 4-fluorophenol at 400 MHz and 600 MHz can be compared. Using the 400 MHz instrument, insufficient ^19^F NMR signal for a measurement was observed from the aqueous phase when 8 transients were applied. However, with just 2 transients using the 600 MHz instrument, a log *P* of 1.74 ± 0.01 could be measured from the same sample (lit. 1.77) with satisfactory signal-to-noise of 35.66 : 1 (Fig. 3).

## Conclusions

Full implementation of this method provides a robust, straightforward and efficient medium-to-high throughput log *P* measurement technique for fluorinated molecules with log *P* values of 3 or lower using a measurement timescale of 1 hour or less with acceptable accuracy (±0.03, maximum deviation from literature of 0.13 log units), when measuring samples of concentration as low as 0.01 M, using a 600 MHz instrument equipped with a H/F-C-N-z helium cryoprobe. When using a 400 MHz instrument, molecules with log *P* of 3 can be measured using samples of 0.34 M in under 2 hours (±0.04, deviation from literature value of 0.09 log units). It has been shown that molecules with a log *P* below 3 are less likely to have issues with toxicity during clinical trials,^33^ meaning this method may be useful in drug discovery campaigns as a simple assay of lipophilicity to monitor structure–property relationships. Molecules of log *P* > 3.0 may also be accessed using a high number of transients per slice. This is an improvement on the analogous ^1^H NMR method in that it does not require a known reference compound, has been demonstrated to measure log *P* beyond 1.66,^26^ and an improvement on the previous ^19^F method in that it does not require a reference or the preparation of multiple samples.^22^ Although limited to molecules containing at least one fluorine atom, this NMR-based approach is an improvement on previously published methods for such molecules for four reasons: first, it is sensitive: the technique allows measurement of a wide range of log *P* values as demonstrated in this article. Second, data acquisition and interpretation are straightforward and require no specialist knowledge other than a good understanding of NMR data integration and some understanding of 1D NMR data manipulation. Third, the data are self-explanatory: the use of ^19^F as the observe nucleus and the simplicity of sample preparation results in very simple NMR spectra suitable for a non-expert to understand. Fourth and finally, no prior knowledge is required of the log *P* value of the analyte and no internal or external standards are required to facilitate the measurement. This technique represents an operationally simple, efficient and broadly applicable method. It enables the measurement of partition coefficients of fluorinated molecules within a range which is relevant to early-stage bioactive molecule development. With a high degree of confidence, the synthetic or medicinal chemist is herein equipped with a method suitable for generating log *P* as a standard piece of readily accessible characterisation data.

## Materials and methods

Reagents were purchased from commercial sources (Fluorochem, Merck, Thermofisher, TCI) and used without further purification.

### Sample preparation

#### Capillary method

0.11–0.21 mmol (for samples with log *P* expected to be between ±2, requiring acquisition times of around 1 hour or greater) neat analyte was added to the NMR tube (by micropipette in the case of analytes which are liquid at room temperature or by spatula for solids) followed by 200 μL of *n*-octanol and 200 μL deionised water, measured by syringe or micropipette. The tube was capped, shaken manually and the phases allowed to separate at room temperature for at least 1 hour (aided by centrifugation where necessary), and a glass capillary containing deuterium oxide was added.

#### No lock method

0.01–0.11 mmol neat analyte was added to the NMR tube followed by 270 μL of *n*-octanol and 270 μL deionised water, measured by syringe or micropipette. The tube was capped, shaken manually and the phases allowed to separate at room temperature for at least 1 hour (aided by centrifugation where necessary).

#### NMR spectroscopy

The NMR tube position was adjusted using a NMR sample depth gauge to ensure that the interface between the two phases was located correctly and coincides with the centre of the radio-frequency (rf) coil of the NMR probe. Standard 1D ^19^F or ^19^F-{^1^H} and slice-selective ^19^F or ^19^F-{^1^H} NMR spectra were acquired under full automation at magnetic field strengths of either 9.4 T (400.13 MHz for ^1^H NMR) or 14.1 T (600.13 MHz for ^1^H NMR) using Bruker Ultrashield NMR magnets at 298 K. Automated ^19^F NMR measurements made at 9.4 T used a 5 mm BBFO-z SmartProbe [iProbe] connected to an AVANCE III Nanobay, two channel Bruker NMR spectrometer running under Topspin version 3.7 and equipped for X-nucleus observation of ^19^F/ ^31^P-^109^Ag and designed with ^1^H capability for decoupling or observation and fitted for automated tuning and matching on both channels. Automated ^19^F NMR measurements made at 14.1 T used a 5 mm H/F-C-N-z helium cryoprobe connected to an AVANCE NEO, five channel Bruker NMR spectrometer running under Topspin version 4.1 and equipped for ^19^F/^1^H observe/decouple among other capabilities and fitted for automated tuning and matching on all channels. Both spectrometers were equipped with automation carousels (SampleCase 60 and SampleCase 24 respectively) and operated under IconNMR for fully automated data acquisition. Prior to partitioned sample insertion on either spectrometer, the magnetic field homogeneity was set by shimming a homogeneous D_2_O sample. For partitioned samples carrying a capillary filled with D_2_O, lock conditions were specifically set to allow for data acquisition with optimal lineshape without lock-related distortions (see SI for further details of lock optimization for D_2_O-filled capillary inserts). Further magnetic field optimization for partitioned samples run under automation was disabled. For partitioned samples with no D_2_O capillary insert, the lock was disengaged (turned off and disabled with lock gain and power set automatically to zero and lock sweep turned off, which occurs when lock = off is set in the acquisition parameters under automation). Parameters were called by automation program “find19Fo1_run_FlogP” (see SI for further details). This allowed the initial setting of a very wide sweep width (490 ppm centred at o1p = −50 ppm). Following this a single pulse, scout scan ^19^F NMR spectrum was acquired, automatically processed and peak picked to select for the largest signal in the spectrum. This signal was then used to set the central offset frequency (o1) for the spatially encoded data acquisitions. The latter were typically acquired as follows.^19^F NMR data acquired without ^1^H decoupling used pulse program selgradgpse2d when data were typically acquired into 16 384 data points over a frequency width equivalent to 10 ppm for an acquisition time of 2.17 s (at magnetic field strength = 9.4 T, c.f. 1.47 s at magnetic field strength = 14.1 T). ^19^F-{^1^H} NMR data (acquired with proton decoupling) used pulse program selgradgpigse2d when data were typically acquired into 2048 data points over a frequency width equivalent to 10 ppm for an acquisition time of 0.27 s (at magnetic field strength = 9.4 T, c.f. 0.184 s at magnetic field strength = 14.1 T), the reduced number of data points and limited data acquisition time being applied to limit the effects of r.f. heating while inverse proton decoupling was applied using decoupler profile Waltz16, centred at ^1^H o2p = 5.0 ppm. In either case, data were typically acquired initially with 16 transients following 2 dummy transients and using a relaxation delay, d1 = 30 s between transients. In each case and for each experiment, two frequency offset, Rsnob selective 180° refocussing pulses were calculated on-the-fly at the time of pulse program execution using the Bruker program Wavemaker, which interprets pulse shape coding from within pulse sequence code. The two pulses were typically calculated with offsets, *Ω*, equal to o1p ± 60 ppm, being stored and called consecutively during the experiment. This consisted of two “increments”, the first selecting a slice from one phase of the sample and the second selecting a slice from the other phase. It should be noted that the offset may be altered if required to suite the magnetic field being used. For instance, at 9.4 T, *Ω* = 60 ppm for ^19^F NMR is equivalent to 22.5 kHz, which converts, under a linear z-field gradient of 16%, to a distance from the solvent/solvent interface, *d*, of 6.4 mm when considered in the context of eqn (2). The equivalent distance when operating at 14.1 T and using exactly the same parameters is 9.6 mm. Increasing the gradient strength to 24% at this field strength restores slice selection to the same distance from the solvent/solvent interface at 6.4 mm.2
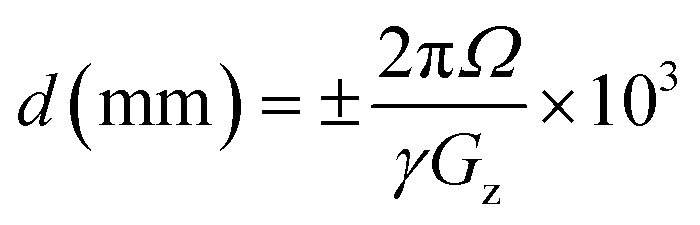
Following data acquisition, the data are automatically processed through a set of scripts, full details of which are provided in the SI along with further details of the evolution of the NMR methods and procedures and instructions for implementing the procedures on equivalent NMR systems to those used in this work.

## Author contributions

Susanna Wood: concept, investigation, analysis, writing – original draft preparation, reviewing & editing. Fiona Gordon: initial investigations & analysis. Robin Stein: revision of methodology, writing – reviewing and editing. Mark Howard: investigation, analysis, writing – reviewing and editing. John Parkinson: methodology, investigation, writing – methodology, SI preparation, original draft preparation, reviewing & editing.

## Conflicts of interest

The authors declare that they have no known competing financial interests or personal relationships that could have appeared to influence the work reported in this paper.

## Supplementary Material

MD-017-D5MD00678C-s001

## Data Availability

Selected data underpinning this publication are openly available from the University of Strathclyde KnowledgeBase at https://doi.org/10.15129/6ca1ffd8-270b-4ede-a3c0-91e083d57e21 and referenced in the SI. Supplementary information is available. See DOI: https://doi.org/10.1039/d5md00678c.
