# Supplementary material for: Efficient log P determination by automated, spatially encoded 19F NMR spectroscopy
Source: RSC Med Chem. 2026 Jan 12;17(3):1550–8. doi: 10.1039/d5md00678c (PMC12836857; doi:10.1039/d5md00678c)
Supplement: MD-017-D5MD00678C-s001 [file MD-017-D5MD00678C-s001.pdf]

Supplementary Information (SI) for RSC Medicinal Chemistry.
This journal is © The Royal Society of Chemistry 2026

Efficient Log*P* determination by automated, spatially encoded ^{19}F NMR Spectroscopy

Susanna H. Wood[a]*, Fiona Gordon[a], Robin S. Stein[b], Mark J. Howard[b] and John A. Parkinson[a]

[a]Department of Pure and Applied Chemistry, University of Strathclyde, Thomas Graham Building, 295 Cathedral Street, Glasgow, G1 1XL, UK

[b]Bruker (UK) Ltd., Welland House, Westwood Business Park, Longwood Close, Coventry CV4 8HZ, UK

SUPPORTING INFORMATION

*Corresponding Author:

Dr. Susanna H. Wood susanna.h.wood@strath.ac.uk

Table of Contents

1.0 Method Development 2
1.1 Principle 2
1.2 Evaluating the extent of deviation of NMR response from ideal 2
1.3 NMR lineshape considerations 7
1.4 n-Octanol/water bilayer samples, lineshape, deuterium lock and deuterium lock adjustment .. 7
1.5 Initial log*P* measurements 10
1.6 Signal-to-noise considerations when measuring log*P* 11
1.7 Log*P* Measurements 12
1.8 Pulse sequences for measuring log*P* by ^{19}F NMR with spatial encoding 17
1.9 Trial procedures and testing 20
1.10 Automated measurement on Bruker AVANCE and AVANCE NEO systems under IconNMR. ...22
1.11 Code 26
2.0 References 42

1.0 Method Development

Development of the final version of the procedure reported in this article was through several stages. For transparency, these stages are described.

1.1 Principle

NMR spectroscopy is quantitative under carefully controlled conditions. The ratio of integrals for NMR signals of a compound partitioned between water and n-octanol within the same sample volume should lead directly to log*P* according to Equation 1. Here, integral ratio is directly related to the ratio of solute in upper (organic) and lower (aqueous) solvent layers with equilibrium partitioning of the solute:

$$logP = log\frac{[Solute\ in\ Octan-1-ol]}{[Solute\ in\ Water]} = log\frac{Integral\ of\ Octan-1-ol\ Layer\ Solute\ NMR\ Signal}{Integral\ of\ Water\ Layer\ Solute\ NMR\ Signal} \quad \text{Eq. 1}$$

For this to hold for absolute quantification, the NMR response to the applied radio frequency (rf) field must be equal in each solvent layer. A radio-frequency pulse or train of pulses should therefore ideally yield the same NMR response in all solute molecules across all solvents within the same NMR tube. For a bilayer sample made of two immiscible solvents, a tuning mismatch occurs due to susceptibility differences between the solvents in use. Such mismatch may alter excitation profiles for solute partitioned into different layers, potentially yielding differences in the resulting integrals and leading to false log*P* values. Assessment of the impact of different samples on NMR probe tuning and signal response was therefore made to understand the potential severity of such differences and inform technique development for general application in an NMR spectroscopy context.

1.2 Evaluating the extent of deviation of NMR response from ideal

NMR data were acquired using a standard delay-90°-acquire pulse sequence using partitioned analytes for which the ^{19}F T_1 longitudinal spin-lattice relaxation time constant for each NMR signal was also measured. This ensured that the larger of any ^{19}F T_1 value was used to set the relaxation delay.

Initially, bilayer test samples were created using deuterated chloroform, $CDCl_3$ (bottom layer) and deuterated water, D_2O (top layer). This combination had two benefits. Firstly, it avoided the need for solvent suppression to be included into the method during early testing stages when ^1H NMR data were also being collected. Secondly, this solvent combination provided a source of deuterium for the NMR spectrometer ^2H field-frequency lock system. $CDCl_3$ and D_2O samples containing the same analyte were also prepared as separate homogeneous NMR samples and analysed independently for reference purposes.

Empirical parameter calibration and setting was achieved using trifluoroethanol, CF_3CH_2OH (selected as the partitioning solute) and trifluorotoluene, $C_6H_5CF_3$ (selected as the layer-labelling, hydrophobic solute, used to indicate when the organic layer was the source of the ^{19}F NMR response (**Figure S1**).

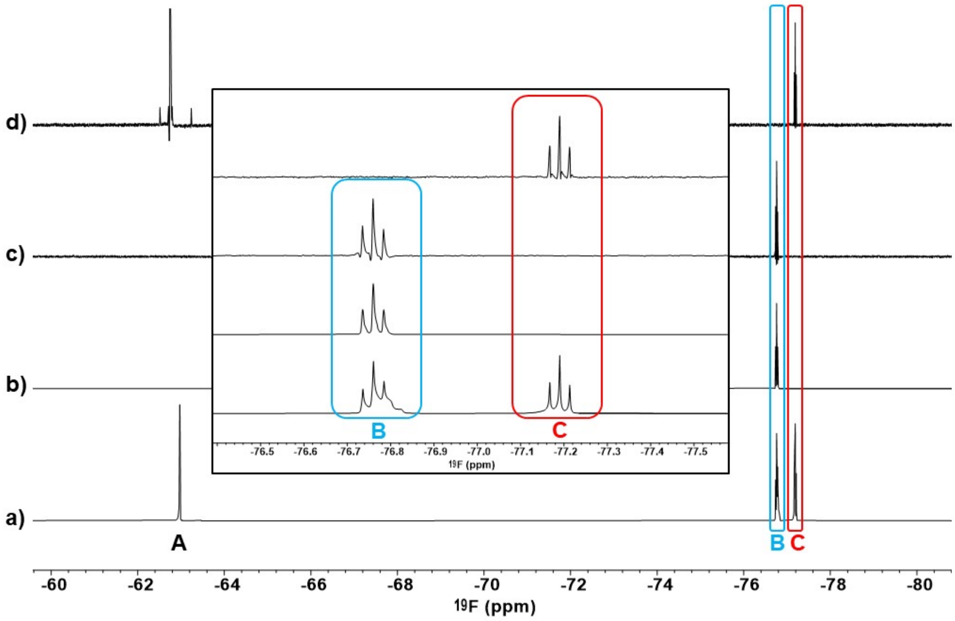

Figure S1. ^{19}F NMR spectra of trifluoroethanol, CF_3CH_2OH (TFE), and trifluorotoluene, $C_6H_5CF_3$ (TFT) showing full NMR spectra with [inset] expansions focussed on the triplet ^{19}F NMR signals from TFE. a) Single pulse-acquire ^{19}F NMR spectrum of layered $CDCl_3/D_2O$ sample containing TFE (**B**, **C**) and TFT (**A**). b) Single pulse-acquire ^{19}F NMR spectrum of a D_2O sample of TFE (**B**). c) ^{19}F NMR spectrum acquired with spatial encoding selecting for upper D_2O layer (**B**). d) ^{19}F NMR spectrum with spatial encoding selecting for lower $CDCl_3$ layer (**C**). **A**: $C_6H_5C\underline{F}_3$ signal from TFT; **B** and boxed: (blue colour code) – $C\underline{F}_3CH_2OH$ ^{19}F NMR signal of TFE from upper D_2O layer; **C** and boxed: (red colour code) – $C\underline{F}_3CH_2OH$ ^{19}F NMR signal of TFE from lower $CDCl_3$ layer. NMR data were acquired at a magnetic field strength of 9.4 T using a two-channel Bruker AVANCE Nanobay NMR spectrometer equipped with a BBFO-z probehead operating at a probehead temperature of 300 K. Acquisition conditions were as follows: 1D ^{19}F single pulse-acquire data were collected into 65536 data points over a frequency width of 30.05 ppm (11312.2 Hz) for an acquisition time of 2.89 s and centred at $\delta^{19}F$ = -70 ppm (offset o1 = -26347.30 Hz). Data were acquired with 16 transients and 2 dummy transients with a relaxation delay of 2.0 s. 1D slice-selective ^{19}F NMR data were acquired using the selective spin-echo pulse sequence shown schematically at **Figure S6a** of this supporting information. Selective excitation 90° (Gaussian 4 Cascade, 1303.33 µs duration) and selective refocussing 180° (Rsnob, 388.67 µs duration) r.f. pulses were applied for a bandwidth of 6kHz over a 16% z-gradient (8.8 G/cm) with a frequency offset (spoffs) of ± 25kHz to select for 1.7 mm thick slices at ± 7 mm either side of the $CDCl_3/D_2O$ solvent interface. Data were acquired with 16 transients and 2 dummy transients over a frequency width equivalent to

30 ppm (11312.2 Hz, acquisition time = 2.89 s) into 65536 data points and centred at δ^{19}F = -70 ppm (o1 = -26348.3 Hz) with an initial relaxation time d1 = 2 s between each transient. These data are available at https://doi.org/10.15129/6ca1ffd8-270b-4ede-a3c0-91e083d57e21.

The expected lower (higher density) $CDCl_3$ layer is distinguished by the presence of the TFT ^{19}F NMR signal in the region δ^{19}F = -62 ppm for the bilayered sample (**Figure S1d**). This signal is absent from the reference D_2O sample ^{19}F NMR spectrum (**Figure S1b**), (since TFT is not in this sample).

These data are notable for two features.

A) Chemical shift difference is observed for resolved ^{19}F NMR signals for the same solute in different solvents for NMR spectra acquired on the bilayer sample (**Figure S1a**). This is evident in a spectrum acquired across the entire length of the detectable volume of the sample using a single pulse-acquire pulse sequence. Simplistically, this approach might be considered suitable for creating data by which log*P* could be readily determined. Realistically, this assumption fails to address several crucial factors including that chemical shift difference may not be significantly distinct or exist at all for the same solute in different solvent layers. Such an observation would complicate or preclude use of an NMR approach for measuring log*P*.

B) Spatial encoding distinguishes distinct layers within the bilayer sample. The example data clearly reveal this, as shown by the presence of the TFT ^{19}F NMR signal at δ^{19}F = - 62 ppm (**Figure S1a** and **Figure S1d**).

Knowledge of the bilayer-origins of each ^{19}F NMR signal in the $CDCl_3$ $(TFE/TFT)_{Lower}/D_2O$ $(TFE)_{Upper}$ bilayer system was a prerequisite for measuring the NMR response, particularly when considering the size of the response *versus* the strength of the applied r.f. field. Parity in the measured response for the solute partitioned into different physical layers of a bilayer sample requires the r.f. pulse response to be determined for the solute in different solvents when the NMR probehead is optimally tuned. Tuning and matching a specific bilayer sample for ^{19}F NMR signal observation is achieved as usual by observing a minimum in the Q-curve of the NMR spectrometer tuning response. For this $CDCl_3/D_2O$ test sample, ^{19}F NMR spectra were subsequently acquired as an array using a single-pulse-acquire pulse sequence. The r.f. pulse length was arrayed from 2 µs to 62 µs in 2 µs steps at 30 W of power and with a 25 s relaxation delay, adjusted to allow for full recovery of spin magnetization when assuming the longest T_1 relaxation time constant to be 5 s. The results (**Figure S2**) showed evidence of differences in the NMR response depending on which solvent layer the ^{19}F NMR signal originated from.

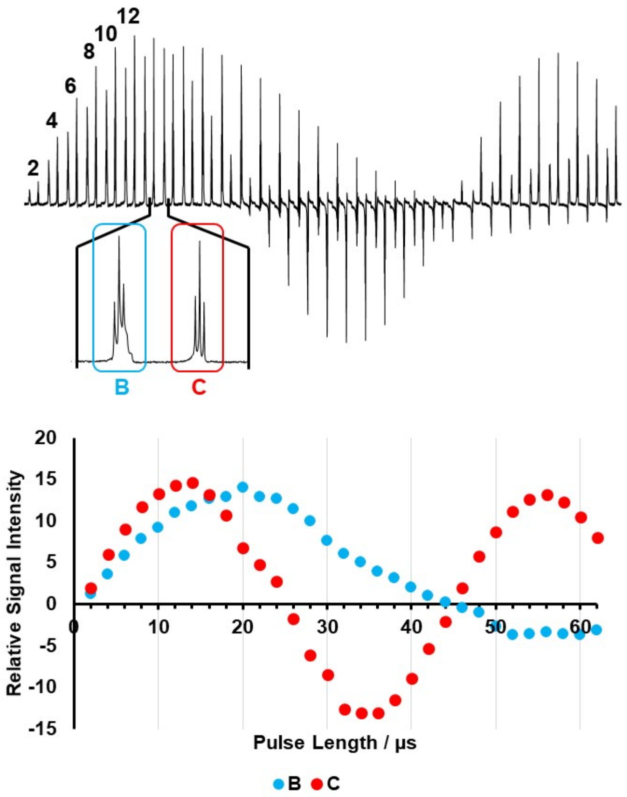

Figure S2: ^{19}F NMR signal response from C<u>F</u>$_3$CH$_2$OH as a function of bilayer solvent. TOP: raw signal response for signal **B** (upper D_2O layer) and signal **C** (lower $CDCl_3$ layer) as described in **Figure S1** and shown expanded with colour coded boxes; the same signals are represented at pulse lengths of 2, 4, 6, 8, 10, 12 etc. μs up to a total of 62 μs in increments of 2 μs. BOTTOM: signal intensities extracted from raw data plotted as a function of pulse length - **red data points** correspond to signal **C**, **blue data points** correspond to signal **B**.

These data show the need for caution. In this instance the lower $CDCl_3$ layer yielded a 90° pulse length of 13 μs at 30 W of power. In contrast, the ^{19}F 90° pulse and maximum signal response achieved for the upper D_2O layer occurred at approximately twice this value and closer to 26 μs at 30W of power. Under these conditions, when the maximum ^{19}F NMR signal response is measured for solute in the lower $CDCl_3$ layer, the intensity of the ^{19}F NMR signal from the same solute in the aqueous layer arises from only a 45° r.f. pulse. The result is detection of only 70% of maximum signal in one layer when 100% of signal is detected in the other layer. A difference like this would significantly affect signal integration and cause such an error in the direct determination of log*P* as to make the method void. The condition suggests a need for the application of a correction factor or recalibration of pulses for each layer with subsequent recording of parallel sets of data for different layers. In principle, the resulting data could then be directly compared. While this was a concern in the method development phase, it would be tedious in practice for general, non-expert application, making the approach less

than accessible for general use. The significant difference in tuning response of $CDCl_3$ compared with D_2O was expected to be largely responsible for this difference. For this reason, it was important to make similar assessment of an n-octanol/water bilayer system to determine whether this behaviour would be a deal breaker or not when considering further use of this approach to log*P* measurement. Similar data to that shown in **Figure S2** but from a typical n-octanol/water bilayer sample is shown in **Figure S3**.

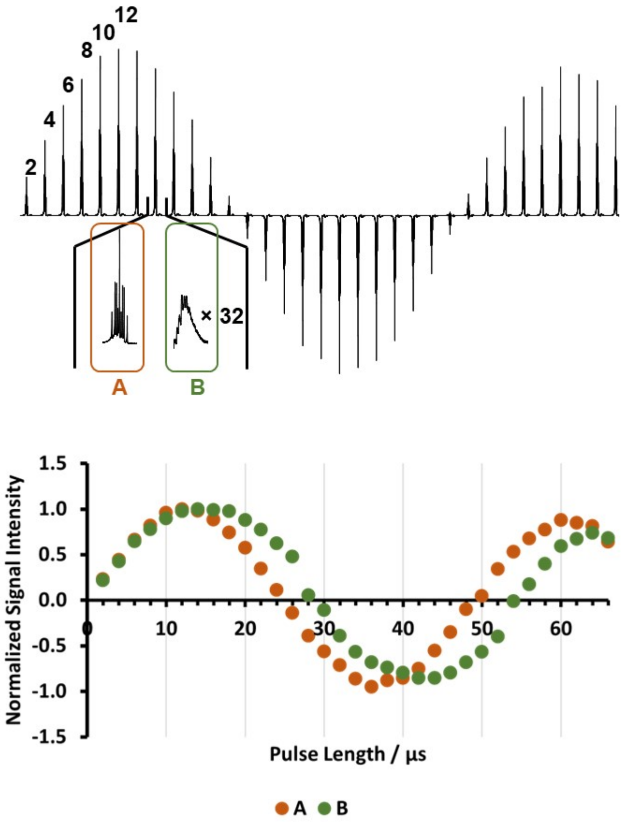

Figure S3: ^{19}F NMR signal responses from Fluoroethanol partitioned between n-octanol and water and acquired at a magnetic field strength of 14.1 T using a -delay-single_pulse-acquire NMR experiment across the whole of a bilayer n-octanol/water sample as a function pulse length. TOP: raw signal response for signal **A** (upper n-octanol layer) and signal **B** (lower aqueous layer) and shown expanded with colour coded boxes; the same signals are represented at pulse lengths of 2, 4, 6, 8, 10, 12 etc. µs up to a total of 66 µs in increments of 2 µs. BOTTOM: Normalized ^{19}F NMR signal intensities extracted from the raw data and plotted as a function of pulse length - **brown data points** correspond to signal **A**, **green data points** correspond to signal **B**.

These measurements revealed a very small difference in the ^{19}F 90° pulse length for responses from n-octanol and water layers of the same sample: ^{19}F pw$_{90}$(n-octanol) = 12.5 µs; ^{19}F pw$_{90}$(water) = 13.0 µs. By calibrating all pulses based on a ^{19}F pw$_{90}$ = 13.0 µs, such a difference would result in a pulse

angle of 86.54° rather than 90°, translating into observation of 99.8% rather than 100% of signal. The propagation of this error to any determined log*P* value would show in the third decimal place. In other words, the effect is negligible when the difference in pulse length experienced by analyte in the upper n-octanol layer compare with that experienced by analyte in the lower aqueous layer is of this order. Care should be taken to check such a response by measuring the ^{19}F 90° pulse length of a suitable sample partitioned between n-octanol/water sample on the NMR spectrometer being used. In our experience of implementing the described procedures on several NMR spectrometers, differences in this value, when operating for instance at either 9.4 T using a BBFO-z SmartProbe [iProbe] or at 14.1 T using a H/F-C-N Helium cryoprobe with n-octanol/water partitioned samples, are so minor as to be negligible for all practical purposes of measuring log*P* using this approach.

1.3 NMR lineshape considerations

Mixed NMR resonance lineshape is apparent within the data. This can be seen by comparing the ^{19}F NMR signal of TFE in the D_2O layer of the bilayer sample (**Figure S1a**, **signal B**, inset) with the same signal for the reference D_2O sample of TFE (**Figure S1b**, **signal B**, inset) and that of the same NMR signal for the spatially encoded ^{19}F NMR spectrum when selecting for the upper D_2O layer (**Figure S1c**, **signal B**, inset). Lineshape is partially a function of magnetic field homogeneity along a sample's length. For a bilayer sample, lineshape distortion as a summation along the whole sample length is anticipated owing to magnetic field inhomogeneity at and approaching the solvent-to-solvent interface. In contrast, the spatially encoded NMR spectrum results from a thin, horizontal sample slice that is distant from the solvent-to-solvent interface, where good to excellent local averaged B_0 homogeneity exists. An advantage of the slice-selective approach is that even when samples are not shimmed (as is the case in this protocol), lineshape is still acceptable in the narrow regions of samples being selected, provided these are distant from the solvent-to-solvent interface. These contrasting data show clearly that integration of signals acquired using a single pulse-acquire approach to NMR data collection for the entire bilayer sample is unsuitable for determining log*P*, thereby underlining the significance of the spatial encoding approach.

1.4 n-Octanol/water bilayer samples, lineshape, deuterium lock and deuterium lock adjustment

The adoption of ^{19}F NMR signal observation for this method arises from the presence of fluorine in many drug molecules and fragment libraries.[1] In contrast to ^{1}H NMR, ^{19}F NMR provides two distinct advantages.

A) The need to handle intense solvent ^{1}H NMR signals arising from the n-octanol/water bilayer system is avoided. Such ^{1}H NMR signals also occupy key regions of the ^{1}H NMR frequency window containing

solute signals, which makes taking a ^{1}H NMR approach to measuring log*P* relatively challenging for fully automated measurements.

B) The generation of background-free ^{19}F NMR data arising from fluorine-labelled solute molecules altogether avoids the need for solvent signal suppression.

Protic solvents (water and n-octanol) mean the absence of deuterium, which is widely used as the source of NMR field/frequency lock. For short duration experiments, this is acceptable. By contrast, long duration experiments (for instance when running samples for extended overnight periods) are subject to magnetic field drift. This generally affects lineshape quality. It is recommended that long duration experiments are carried out on samples that host a sealed capillary containing D_2O to provide the source for an NMR field/frequency lock signal. Such an approach was validated in this work on a mixture of trifluoroethanol, CF_3CH_2OH (TFE) and hexafluoroisopropanol, $(CF_3)_2CHOH$ (HFIP) for which experimental log*P* values are also known.[2] Spectra from initial studies of this partitioned sample (between n-octanol and water) (**Figure S4**) show some notable features worthy of comment.

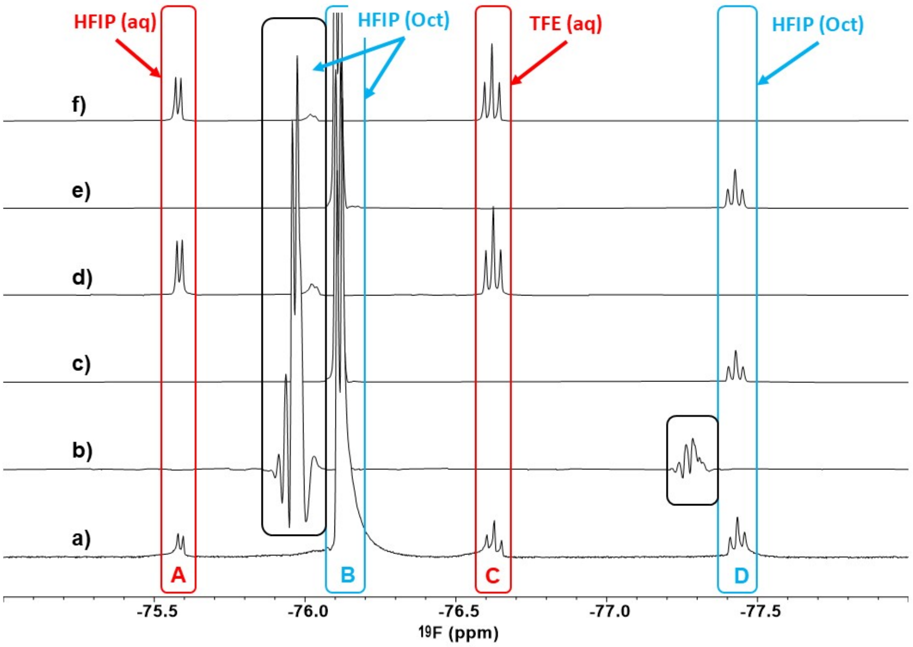

Figure S4: ^{19}F NMR spectra from TFE and HFIP partitioned between n-octanol and water with a D_2O capillary insert. **a)** Single-pulse-acquire spectrum across the entire sample length with sample locked under automated lock conditions. **b)** With spatial encoding and n-octanol layer selection. The deuterium lock is applied using standard parameters for D_2O. The data are displaced horizontally to high frequency by 0.2 ppm with respect to the axis displayed to clearly show signal B but in the **black box**. **c)** With spatial encoding and n-octanol layer selected but with sample unlocked. **d)** With spatial encoding, aqueous layer selected and sample unlocked. **e)** With spatial encoding, n-octanol layer

selected and sample locked with lock parameters adjusted for the capillary in the mixed solvent system. **f)** With spatial encoding, aqueous layer selection and sample locked using optimised lock parameters. See caption to **Figure S1** for experimental details.

The spectrum for the whole sample (**Figure S4a**) shows four signals (two doublets **A** and **B**, and two triplets, **C** and **D**). These arise from two different solute molecules partitioned into two solvent layers. The sample was tuned to ^{19}F but not shimmed following shimming on a homogeneous D_2O sample run immediately prior to insertion of the bilayer sample into the NMR magnet. With deuterium lock applied, the spatially encoded NMR spectrum on the upper n-octanol layer shows significant lineshape distortion (**Figure S4b**). Association of this lineshape distortion with lock response following application of pulsed field gradients is clear from data acquired when the deuterium lock sweep is switched off and lock power is reduced to zero (**Figures S4c, S4d)**. Systematic adjustment of lock loop filter, lock loop gain and lock loop time, carried out to assess their impact on the ^{19}F NMR lineshape, indicated that reduction of the loop gain value by 20 units from the value read automatically by the spectrometer under automated locking gives acceptable, reproducible ^{19}F NMR lineshape for locked samples (**Figure 4e** and **4f**). The effects encountered for long duration experiments without field frequency lock are shown for comparison (**Figures S5**).

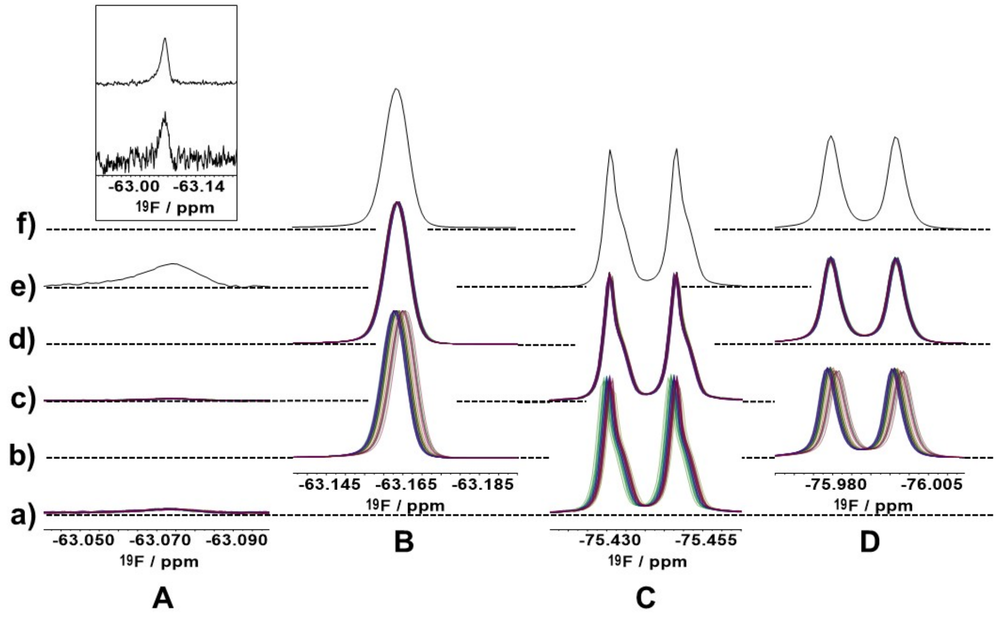

Figure S5: Long duration ^{19}F NMR data acquisition with spatial encoding for unlocked n-octanol/water bilayer sample of trifluorotoluene, TFT, $CF_3C_6H_5$, and hexafluoroisopropanol, HFIP, $(CF_3)_2CHOH$. a) superposition of 32 separate ^{19}F NMR spectra each acquired with 128 transients, with spatial encoding selecting for lower, aqueous layer; b) as for a) but with spatial encoding selecting for upper n-octanol layer; c) as for a) but following data alignment and removal of outlier spectra

containing degraded signal lineshape; d) as for b) following similar data alignment and equivalent data removal to match the same number of spectra for each sample layer; e) summation of aligned spectra for lower aqueous layer; f) summation of aligned spectra for upper n-octanol layer. **A** - Aqueous layer ^{19}F NMR signal from TFT; **B** – n-Octanol layer ^{19}F NMR signal from TFT; **C** - Aqueous layer ^{19}F NMR signal from HFIP; **D** – n-Octanol layer ^{19}F NMR signal from HFIP. Inset: Aqueous layer ^{19}F NMR signal from TFT from 128 transients [lower] and 128 × 26 transients [upper] following alignment and summation of separate, 128 transient ^{19}F NMR spectra. NMR data were acquired at a magnetic field strength of 9.4 T using a two-channel Bruker AVANCE Nanobay NMR spectrometer equipped with a BBFO-z probehead operating at a temperature of 300 K. For experimental details see caption to **Figure S1** and data availability at https://doi.org/10.15129/6ca1ffd8-270b-4ede-a3c0-91e083d57e21

1.5 Initial log*P* measurements

The method was initially operated under manual control of the instrument, allowing validation of the technique in the measurement of a number of fluorinated molecules as shown in **Figure S6**, below.

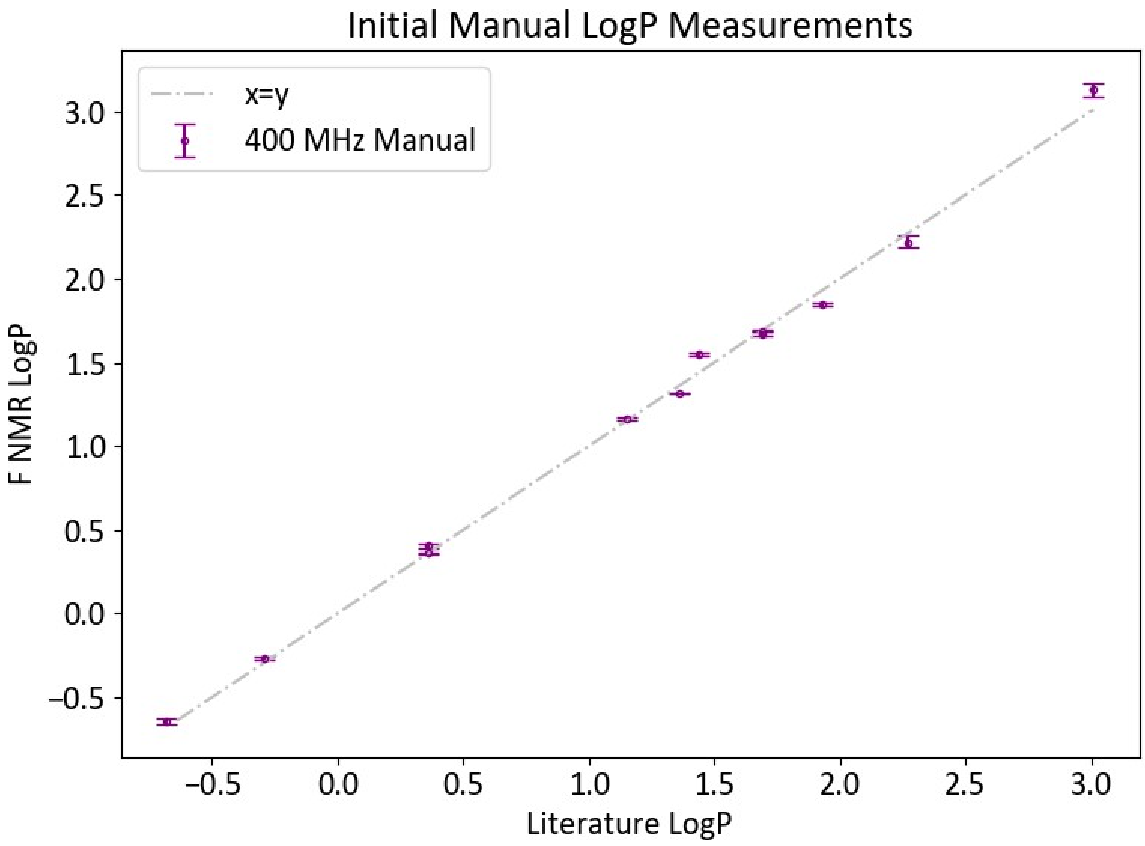

Figure S6: Results of log*P* measurements under manual control.

1.6 Signal-to-noise considerations when measuring log*P*

The stepwise data acquisition described by **Figure S5** led to an assessment of the effects of signal-to-noise on the outcome of log*P* determination. In the context of a significantly different distribution of molecules between solvents, the resulting NMR signal from the most soluble context will have excellent signal-to-noise in contrast to very low signal-to-noise where solute has a low uptake. By aligning and summing different numbers of spectra together from the data reported by **Figure S5**, it was possible to see the effects of improved signal-to-noise on the fit of calculated log*P* with reported values (**Table S1**).

Table S1: log*P* for trifluorotoluene, TFT, as a function of ^{19}F signal-to-noise.

No. Spectra[a]	NS	n-Octanol Layer S/N[b]	Water Layer S/N[b]	Integral n-octanol Layer	Integral Water Layer	log*P*
1	128	6984.89	2.98	99.922	0.079	3.11
4	512	13246.92	6.17	99.924	0.076	3.12
16	2048	27390.06	10.99	99.900	0.010	3.00

[a] ^{19}F NMR spectra were acquired unlocked in blocks of 128 transients using a relaxation delay of 30 s between transients. Other acquisition parameters and conditions are as described previously. Data from each layer were collected separately by frequency offsetting the slice-selective pulses according to the method described in the article text for manual data acquisition. Data from separate layers were processed with 0.3 Hz line broadening and combined by summation from which integrals were measured.

[b] Signal region for the upper n-octanol layer was defined as $\delta^{19}F$ = -63.05 to -63.25 ppm ($\Delta\delta$ = 0.2ppm); signal region of the lower aqueous layer was defined as $\delta^{19}F$ = -63.00 to -63.20 ppm ($\Delta\delta$ = 0.2ppm); noise region was defined as $\delta^{19}F$ = -69.00 to -70.00 ppm (($\Delta\delta$ = 1.0 ppm).

The prepared sample was of relatively low concentration (0.05 M) and therefore used to test the limits of sensitivity and the effect this had on the outcome of log*P* determination. By combining spectra, it is possible to see the effects of how doubling the signal-to-noise influences log*P*. With just 128 transients giving a weak response from the ^{19}F signal from TFT in lower aqueous layer (**Figure S5** inset, lower spectrum), the outcome yields log*P* = 3.11. Doubling the signal-to-noise has little effect on this number and only when a further doubling of the signal-to-noise occurs to yield a value of > 10:1 does the log*P* value come into alignment with the reported literature value of log*P* = 3.01. This sets a minimum threshold for signal-to-noise as around 10:1 as a foundation for more accurate determination of log*P* using this approach.

1.7 Log*P* Measurements

The log*P* for each measurement was determined and compared by applying a consistent approach to the processing and integration of the NMR spectral data. Line broadening of 5 Hz was applied, the baseline was corrected using TopSpin's automated baseline correction function "abs", each signal was integrated across a 1 ppm chemical shift range, with the signal centred in each case, and the log*P* calculated from the integral ratio. The error from the measurement was propagated from the signal-to-noise ratio by calculating the maximum and minimum integral associated with this signal-to-noise followed by calculation of the maximum and minimum log*P* values which could be calculated from these integrals. The results are shown in **Table S2**. The effect of integrating each spectrum separately was compared to integration of the summed spectrum. Integrating each spectrum individually and recording the absolute integrals typically gave advantages in the signal-to-noise, in turn improving confidence in the measurement. Log*P* derived from integration of the summed spectra (setting the water phase integral to 1 and calculating the log*P* from the relative integrals) typically deviated from those calculated by integrating the spectra separately by a maximum of 0.05 log units, provided the signal-to-noise in the summed spectrum was above 10:1. The spectra associated with the data presented in **Table S2** are available for download from: https://doi.org/10.15129/6ca1ffd8-270b-4ede-a3c0-91e083d57e21

Table S2: Log*P* determination by ^{19}F NMR and associated Signal-to-Noise

Compound	mmol	total vol (mL)	Total Conc. (M)	Instrument/ probe	NS/ expt. time[a]	Integral Water Layer	Integral n-Octanol Layer	Water Layer S/N	n-Octanol Layer S/N	Lit. log*P*	Meas. log*P*	Δlit.	Error (+)	Error (-)
4-Fluoro-aniline	0.104	0.54	0.193	400 MHz/ BBFO-z	28/ 00:30:17	4106038	6.2E+07	93.06	3163.2	1.15[3]	1.181	0.031	0.005	0.005
4-Fluoro-aniline	0.104	0.54	0.193	400 MHz/ BBFO-z	28/ 00:30:17	1	15.5083	70.32	2446	1.15[3]	1.191	0.041	0.006	0.006
4-Fluoro-aniline	0.104	0.54	0.193	600 MHz/He cryo	2/ 00:04:02	3.4E+10	4.8E+11	518.73	9045.51	1.15[3]	1.147	-0.003	0.001	0.001
4-Fluoro-aniline	0.104	0.54	0.193	600 MHz/ He cryo	2/ 00:04:02	1	14.1219	408.04	7363.6	1.15[3]	1.150	0.000	0.001	0.001
4-Fluoro-phenylacetic acid	0.106	0.54	0.196	400 MHz/ BBFO-z	28/ 00:30:17	1720713	6.5E+07	40.27	3158.53	1.44[3]	1.579	0.139	0.011	0.011
4-Fluoro-phenylacetic acid	0.106	0.54	0.196	400 MHz/ BBFO-z	28/ 00:30:17	1	39.0627	27.17	2265.1	1.44[3]	1.592	0.152	0.016	0.016
4-Fluoro-phenylacetic acid	0.106	0.54	0.196	600 MHz/ He cryo	2/ 00:04:02	1.5E+10	5E+11	226.79	10928.8	1.44[3]	1.526	0.086	0.002	0.002
4-Fluoro-phenylacetic acid	0.106	0.54	0.196	600 MHz/ He cryo	2/ 00:04:02	1	33.8807	161.85	8018.98	1.44[3]	1.530	0.090	0.003	0.003
3-Fluoro-phenol	0.116	0.40	0.290	400 MHz/ BBFO-z	64/ 01:06:39	2485617	1.9E+08	90.98	4670.98	1.93[3]	1.883	-0.047	0.005	0.005
3-Fluoro-phenol	0.116	0.40	0.290	400 MHz/ BBFO-z	64/ 01:06:39	1	73.3776	66.91	3519.23	1.93[3]	1.866	-0.064	0.007	0.007
3-Fluoro-phenol	0.116	0.40	0.290	600 MHz/ He cryo	2/ 00:04:02	1E+10	6.5E+11	244	14864	1.93[3]	1.805	-0.125	0.002	0.002
3-Fluoro-	0.116	0.40	0.290	600 MHz/	2/	1	63.7972	53.36	2901.13	1.93[3]	1.805	-0.125	0.008	0.008

phenol				He cryo	00:04:02									
Trifluoro-toluene	0.135	0.40	0.339	400 MHz/ BBFO-z	96/ 01:43:11	667853	9.2E+08	14.64	20483.8	3.01[4]	3.139	0.129	0.031	0.029
Trifluoro-toluene	0.135	0.40	0.339	400 MHz/ BBFO-z	96/ 01:43:11	1	1261.48	10.46	14593.8	3.01[4]	3.101	0.091	0.044	0.040
Trifluoro-toluene	0.135	0.40	0.339	600 MHz/ He cryo	2/ 00:04:12	8.7E+08	8.5E+11	21.06	17812.8	3.01[4]	2.990	-0.020	0.021	0.020
Trifluoro-toluene	0.135	0.40	0.339	600 MHz/ He cryo	2/ 00:04:12	1	1074.09	11.14	11533.9	3.01[4]	3.031	0.021	0.041	0.037
Trifluoro-toluene	0.013	0.54	0.024	600 MHz/ He cryo	32/ 00:34:20	2.7E+09	2.7E+12	16.84	15027.1	3.01[4]	2.999	-0.011	0.027	0.025
Trifluoro-toluene	0.013	0.54	0.024	600 MHz/ He cryo	32/ 00:34:20	1	1106.75	11.21	9753.56	3.01[4]	3.044	0.034	0.041	0.037
Fluoro-ethanol	0.097	0.54	0.179	400 MHz/ BBFO-z	10/ 00:12:07	1.4E+07	2800751	470.73	234.36	-0.68[2]	-0.714	-0.034	0.003	0.003
Fluoro-ethanol	0.097	0.54	0.179	400 MHz/ BBFO-z	10/ 00:12:07	1	0.1854	407.66	173.81	-0.68[2]	-0.732	-0.052	0.004	0.004
Fluoro-ethanol	0.097	0.54	0.179	600 MHz/ He cryo	2/ 00:04:12	3.7E+11	6.6E+10	5593.92	1398.95	-0.68[2]	-0.752	-0.072	0.000	0.000
Fluoro-ethanol	0.097	0.54	0.179	600 MHz/ He cryo	2/ 00:04:12	1	0.1759	4166.42	811.98	-0.68[2]	-0.755	-0.075	0.001	0.001
Fluoro-benzene	0.210	0.40	0.525	400 MHz/ BBFO-z	64/ 01:06:39	1509686	2.8E+08	44.34	10605.5	2.27[3]	2.263	-0.007	0.010	0.010
Fluoro-benzene	0.210	0.40	0.525	400 MHz/ BBFO-z	64/ 01:06:39	1	178.187	35.48	9054.24	2.27[3]	2.251	-0.019	0.012	0.012
Fluoro-benzene	0.210	0.40	0.525	600 MHz/ He cryo	2/ 00:04:02	4.8E+09	8.6E+11	120.46	14353.7	2.27[3]	2.249	-0.021	0.004	0.004
Fluoro-benzene	0.210	0.40	0.525	600 MHz/ He cryo	2/ 00:04:02	1	167.76	87.69	9306.58	2.27[3]	2.225	-0.045	0.005	0.005
4-Fluoro-benzyl alcohol	0.105	0.54	0.195	400 MHz/ BBFO-z	28/ 00:30:17	2653372	6.2E+07	71.54	3261.66	1.36[5]	1.369	0.009	0.006	0.006

4-Fluoro-benzyl alcohol	0.105	0.54	0.195	400 MHz/ BBFO-z	28/ 00:30:17	1	22.2259	47.34	2115.15	1.36[5]	1.347	-0.013	0.009	0.009
4-Fluoro-benzyl alcohol	0.105	0.54	0.195	600 MHz/He cryo	2/ 00:04:02	2.3E+10	4.9E+11	434.81	10883.2	1.36[5]	1.320	-0.040	0.001	0.001
4-Fluoro-benzyl alcohol	0.105	0.54	0.195	600 MHz/He cryo	2/ 00:04:02	1	19.9603	308.65	7817.24	1.36[5]	1.300	-0.060	0.001	0.001
Trifluoro-ethanol	0.106	0.54	0.196	400 MHz/BBFO-z	10/ 00:12:07	1.9E+07	4.5E+07	1007.1	2750.21	0.36[2]	0.369	0.009	0.001	0.001
Trifluoro-ethanol	0.106	0.54	0.196	400 MHz/BBFO-z	10/ 00:12:07	1	2.3428	631.47	2259.46	0.36[2]	0.370	0.010	0.001	0.001
Trifluoro-ethanol	0.106	0.54	0.196	600 MHz/He cryo	2/ 00:04:02	4.6E+11	1E+12	10129.1	20045.4	0.36[2]	0.333	-0.027	0.000	0.000
Trifluoro-ethanol	0.106	0.54	0.196	600 MHz/He cryo	2/ 00:04:02	1	2.1418	7069.06	16977.2	0.36[2]	0.331	-0.029	0.000	0.000
Hexafluoro-isopropanol	0.084	0.54	0.155	400 MHz/BBFO-z	10/ 00:12:07	1587281	7E+07	58.51	4602.26	1.67[2]	1.648	-0.022	0.008	0.007
Hexafluoro-isopropanol	0.084	0.54	0.155	400 MHz/BBFO-z	10/ 00:12:07	1	44.3177	43.14	3174.14	1.67[2]	1.647	-0.023	0.010	0.010
Hexafluoro-isopropanol	0.084	0.54	0.155	600 MHz/He cryo	2/ 00:04:02	4.1E+10	1.6E+12	783.31	29082.4	1.67[2]	1.586	-0.084	0.001	0.001
Hexafluoro-isopropanol	0.084	0.54	0.155	600 MHz/He cryo	2/ 00:04:02	1	37.7326	457.58	19508.5	1.67[2]	1.577	-0.093	0.001	0.001
Difluoro-ethanol	0.086	0.54	0.160	400 MHz/BBFO-z	10/ 00:12:07	2.1E+07	1.2E+07	782.85	792.05	-0.29[2]	-0.263	0.027	0.001	0.001

Difluoro-ethanol	0.086	0.54	0.160	400 MHz/BBFO-z	10/ 00:12:07	1	0.5441	592.86	557.69	-0.29[2]	-0.264	0.026	0.002	0.002
Difluoro-ethanol	0.086	0.54	0.160	600 MHz/He cryo	2/ 00:04:02	5.2E+11	2.7E+11	8174.29	5544.35	-0.29[2]	-0.290	0.000	0.000	0.000
Difluoro-ethanol	0.086	0.54	0.160	600 MHz/He cryo	2/ 00:04:02	1	0.5164	3525.57	4831.44	-0.29[2]	-0.287	0.003	0.000	0.000
4-Fluoro-phenol	0.025	0.54	0.046	400 MHz/BBFO-z	64/ 01:06:39	534503	3.2E+07	8.01	1231.33	1.77[3]	1.781	0.011	0.058	0.051
4-Fluoro-phenol	0.025	0.54	0.046	400 MHz/BBFO-z	64/ 01:06:39	1	47.4213	6.73	845.26	1.77[3]	1.676	-0.094	0.070	0.061
4-Fluoro-phenol	0.025	0.54	0.046	600 MHz/He cryo	2/ 00:04:02	1.9E+09	1.1E+11	51.04	1895.77	1.77[3]	1.785	0.015	0.009	0.009
4-Fluoro-phenol	0.025	0.54	0.046	600 MHz/He cryo	2/ 00:04:02	1	55.5844	35.66	1252.77	1.77[3]	1.74495	-0.025	0.013	0.012

[a]Experiment times to acquire the reported data are presented, the number of scans was varied leading to the different experiment times shown. In comparison to the method reported by Linclau *et al.,*[2] which involves sampling the aqueous and organic phases of a stirred biphasic n-octanol/water mixture which contains the compound of interest along with a known reference sample and acquiring ^{19}F NMR spectra of each sample in turn, the recommended NMR parameters in the paper (30s relaxation delay for n-octanol sample, 60s relaxation delay for water sample, 64 transients for each sample) would result in a total NMR experiment time of at least 1.5 hours. The slice selective ^{1}H method reported by Ben-Tal *et al.*[6] used 4 transients per phase, with a 60s relaxation delay (at least 8 minutes) to measure samples of log*P* -1.35-1.66. However, they do not state the sample concentrations and were unable to measure any signal from the n-octanol phase of a molecule with log*P* -2.78 when applying 256 transients to each phase (at least 8.5 hours instrument time).

1.8 Pulse sequences for measuring log*P* by ^{19}F NMR with spatial encoding

Several pulse sequences were explored for spatially encoded ^{19}F NMR measurements for log*P* determination (**Figure S7**). The starting basis was a previous report of ^{1}H NMR studies, which demonstrated utility with an n-octanol/water bilayer system.[7] In this ^{1}H NMR work, the author recommends using a slice-selective spin-echo using slice-selective 90° and 180° pulses as referred to in the main text of this article, which reportedly yielded cleaner NMR data free from artefacts when compared with using a slice-selective 90° pulse only. Our experience agreed with this assessment and in the final stage of our method development, pulse sequence modification was applied to reduce complexity of handling shaped pulses in the context of a fully automated method by using a hard 90° followed by selective 180° degree pulse in the context of a spin-echo. The former approach was used initially; the latter approach was adopted as standard and for which the pulse sequence codes are provided here.

Spatial encoding was initially tested using ^{19}F observation only (^{1}H decoupling not included, **Figure S7a**). Using the Bruker "popt" routine for arraying parameters, pulse offset frequencies, spoffs, for both shaped pulses were arrayed across a series of values ranging from -21500 Hz to +21500 Hz in steps of 3 kHz, allowing sample positioning to be checked. At a magnetic field strength of 9.4 T, this gives rises to measurement across a physical distance of 12 mm ($\pm$ 6 mm either side of the centre of the r.f. coil). Details of pulses are described in the methods section of the main article but using pulse bandwidths of 6 kHz combined with frequency offsets as described and gradient pulses set to 16% of maximum yielded maximum excitation offsets of 6.1 mm from the centre of the receiving coil with a slice thickness of 1.7 mm. Arraying the pulse offset in steps of 3 kHz allowed slices to overlap by half a slice thickness per offset frequency increment. Running the acquisition as a pseudo two-dimensional data array ensured no physical gaps occurred when scanning the length of the NMR sample for signal. This approach ensures alignment of the solvent interface with the rf receiver coil centre when initial set up is carried out (**Figure S8**). Clear differences in lineshape are observed when comparing data acquired either remote from or adjacent to the solvent interface. Good lineshape is evidenced at locations remote from the solvent interface (**Figure S8b** and **S8d**) with signals showing their expected $^{3}J_{FH}$ couplings and splitting patterns, indicating good magnetic field homogeneity over the narrow 1.7 mm range of the separately sampled slices. Sampling adjacent to the solvent interface (**Figure S8c**) yields spectra with substantial lineshape broadening, a result of poor magnetic field homogeneity at the solvent-to-solvent interface. This effect persists for some distance either side of the solvent interface, evidenced through chemical shift displacement and underlying resonance broadening (**Figure S8a**). As described in the main article, adjustment of slice position and thickness can be readily

calculated and should be set when implementing the described procedures for the first time on any NMR spectrometer.

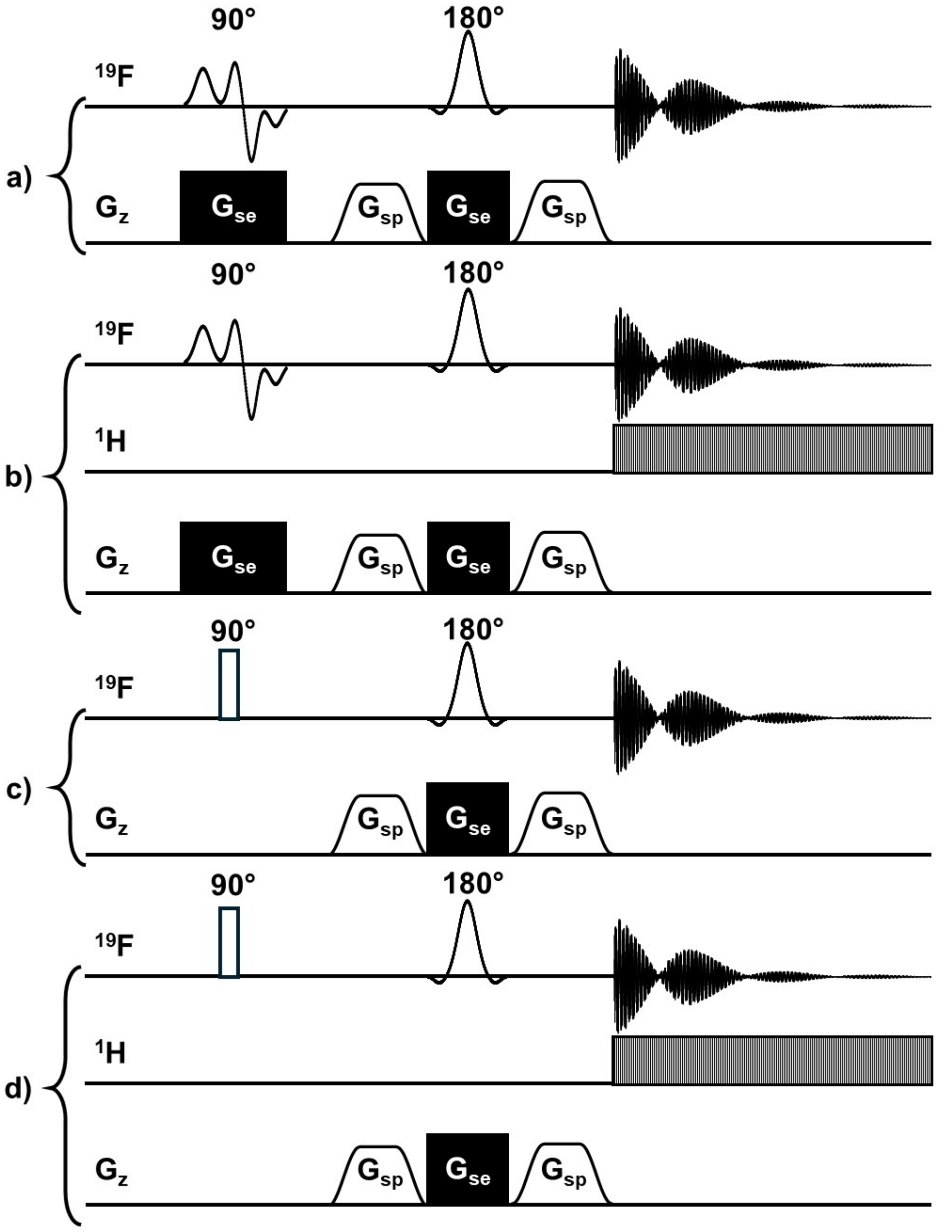

Figure S7: Spatial encoding spin-echo pulse sequences as a basis for automated log*P* determination by ^{19}F NMR spectroscopy. a) ^{19}F observation only using two slice-selective pulses. b) as for a) but with proton decoupling *via* inverse gating. c) ^{19}F observation only, using non-selective hard 90° pulse excitation followed by slice-selective 180° pulse (spatial encoding by gradient pulses, pulse sequence selgradgpse2d in section 1.11). d) as for c) but with proton decoupling *via* inverse gating (pulse sequence selgradgpigse2d in section 1.11). G_z – pulsed field gradients: G_{se}: spatial encoding square gradient = 16% of maximum ≡ 8.8 G cm^{-1}; G_{sp}: spoiling, smoothed square gradient = 45% ≡ 24.8 G cm^{-1}. Shaped excitation (90°) and spin-echo refocussing (180°) r.f. pulses use gaussian cascade and rsnob

profiles respectively: excitation 90° G4 cascade = 1303.33 µs, bandwidth 6 kHz; refocussing 180° Rsnob = 388.67 µs, bandwidth 6 kHz. Pulse powers were initially set manually based on calculations using the Bruker shaped tool module within Topspin.

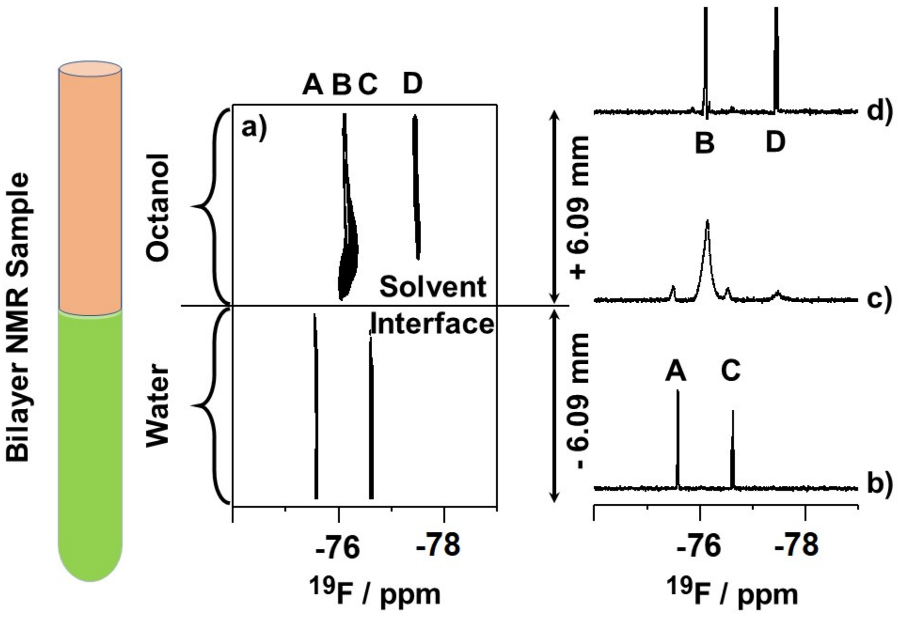

Figure S8: Slice selective ^{19}F NMR data arrayed along a sample length using parameters as described. a) Pseudo 2D data showing ^{19}F NMR responses across 12 mm of the sample length either side of and showing alignment with the solvent interface at the centre of the receiver coil. **A/B**: doublet signals arising from hexafluoroisopropanol, HFIP; **C/D**: triplet signals arising from trifluoroethanol, TFE. b) 1D ^{19}F spatially encoded spectrum at the lower measurement extremity extracted from the pseudo 2D data; c) as b) but at the solvent interface; d) as b) and c) but at the higher measurement extremity. The offset frequency, spoffs, of the selective pulse was varied as -21500 Hz to +21500 Hz using the Bruker "popt" routine for parameter arrays. Data were stored as a serial (ser) file and were transformed along the acquisition dimension only with individual slices separately phase corrected to adjust for the phase modification arising from the pulse offset with spatial encoding. NMR data were acquired at a magnetic field strength of 9.4 T using a two-channel Bruker AVANCE Nanobay NMR spectrometer equipped with a BBFO-z probehead operating at a temperature of 300 K and according to the parameters described in the text. Data are available to download from https://doi.org/10.15129/6ca1ffd8-270b-4ede-a3c0-91e083d57e21

As these data show, optimal line-shape is achieved at a distance of at least 6 mm from the r.f. receiver coil centre. For automation, adjustment of the solvent interface position should be carried out with

care when preparing samples. With solvent interface correctly aligned using a typical NMR sample depth gauge, parameters can be set to ensure that quantitative, spatially encoded NMR data are acquired at a suitable distance from the solvent interface. This ensures optimal NMR line-shape integrity, reproducibility and reliability. These checks are recommended but are not essential provided solvent volumes and correct sample depth gauging guidelines detailed in this article are followed.

1.9 Trial procedures and testing

Definitive automated method development from which reliable log*P* NMR data could be generated automatically with assurance was based on use of the pulse sequences shown at **Figures S7c** and **S7d** for which code is reported. In this work, for long duration experiments, a 2 mm diameter sealed capillary tube containing D_2O for the lock signal was included in the bilayer sample. This required n-octanol and water layer volumes of 200 µL each to fix the solvent interface exactly at the rf receiver coil centre when a 5 mm diameter NMR tube was correctly depth adjusted using the Bruker supplied depth gauge and sample shuttle combination. In the absence of a sealed capillary (for unlocked data acquisition), 270 µL of each solvent is required to accurately set the solvent interface at the rf receiver coil centre.

Walk-up use running under Bruker's IconNMR interface relies on the ability to define lists of shaped pulses in combination with the Wavemaker function of TopSpin. This enables automated generation of complex waveforms without the need for user intervention. Acquisition of data occurs in a pseudo 2D format by incrementing the shaped pulse list to yield two spatially encoded ^{19}F NMR spectra stored in the same serial file as one another and which represent sampling of upper and lower layers of the bilayer sample. Definition of the shaped pulse using the Wavemaker format ensures that a bandwidth of 6 kHz is used for the selective refocussing pulse at frequency offsets yielding 1.7 mm thick sample slices at a distance 6 mm either side of the rf coil centre. The frequency offset may be set as a variable using cnst52 and cnst53. At 9.4 T, setting cnst52 to -60 ppm and cnst53 to + 60 ppm results in slice-selection at $\pm$ 6.1 mm either side of the r.f. coil centre when a 16% gradient pulse accompanies the 6 kHz bandwidth selective Rsnob refocussing pulse. Relaxation delay and the number of transients may be set to ensure that full recovery of spin magnetization occurs between transients and that adequate signal-to-noise ratio is generated for the weakest signals, respectively. These conditions are designed to reduce integration errors and improve the reliability of log*P* values determined from such data. Example data, one using a ^{19}F observe approach and one using ^{19}F observe with inverse gated ^{1}H decoupling are shown following (**Figure S9**).

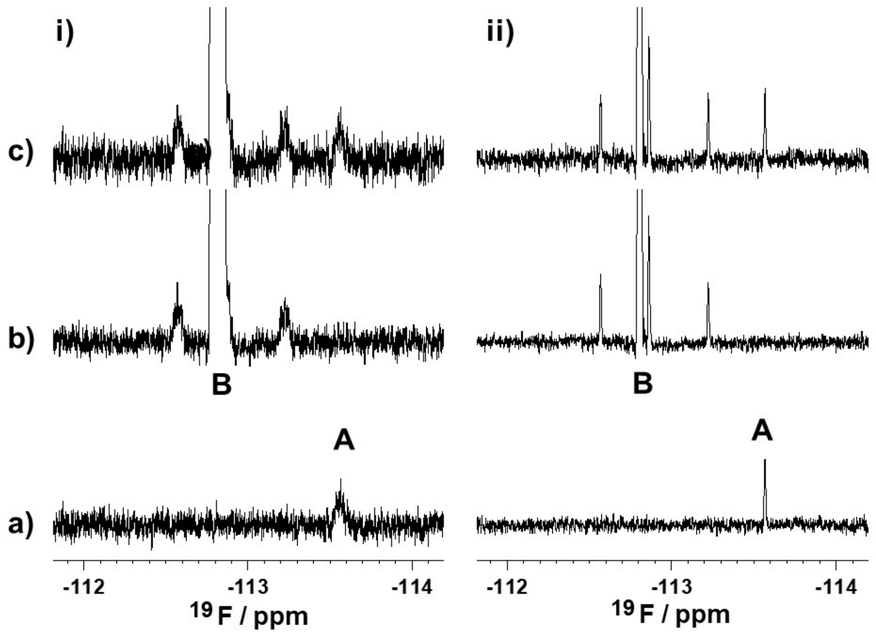

Figure S9: Log*P* by spatially encoded ^{19}F NMR spectroscopy. i) In the absence of ^{1}H-decoupling. ii) Equivalent ^{19}F-{^{1}H} NMR data for the same sample. a) Spectra from lower, aqueous layer. b) Spectra from upper, n-octanol layer. c) Summation of spectra at a) and b). Spectra were acquired to determine, by ^{19}F NMR, the experimental log*P* value for fluorobenzene. **A** - ^{19}F NMR signal from fluorine in fluorobenzene partitioned into the aqueous layer. **B** - as **A** but for the majority fluorobenzene present in the organic, n-octanol layer.

In this example, data were acquired at a B_0 field strength of 9.4 T using a Bruker BBFO-z probe with 256 transients for fluorine NMR data from both upper and lower layers of the bilayer sample and a recycle delay of 25 s per transient, leading to a total experiment time of approximately 4.5 hrs. Individual, slice-selective ^{19}F NMR spectra may be checked for data quality, as shown at **Figure S9a** and **Figure S9b**. Preference is made for integration of the data using the sum of slices spectra (**Figure S9c**). This ensures the same scaling is used for all integrated peaks. For overlapping peaks, the summation may include offsetting the chemical shift scale of one spectrum relative to the other to avoid coincidence of signals arising from solute in different layers of the bilayer sample. The presence of ^{13}C satellites (from $^{1}J_{FC}$ and $^{2}J_{FC}$) should be included in the integration. The log*P* value calculated from these data are consistent with previously published experimentally measured values.[3,4,5,6] The improvement shown in the signal-to-noise ratio for the ^{19}F-{^{1}H} NMR data (**Figure S9ii)**) compared with the equivalent ^{19}F NMR data without proton decoupling (**Figure S9i)**) makes the former approach desirable when the NMR spectrometer hardware allows. Under these conditions, care should be taken to reduce the acquisition time, as described in the main article, to prevent excessive strain on the r.f. coils and avoid r.f. sample heating. With judicious use of processing parameters, log*P* determination

by spatially encoded ^{19}F NMR data acquired without proton decoupling is feasible if this is the only available hardware configuration available to the experimentalist.

Several approaches for improving signal-to-noise may considered. Spatial encoding itself reduces the quantity of detectable signal overall. However, this may be enhanced through acquisition of data in which multiple frequency offsets are encoded into each pulse, thereby enabling summation of individual slices over a bigger physical volume. Care is required to ensure that lineshape integrity is maintained across separate slices within the same sample layer or that sampling does not occur close to the solvent-to-solvent interface, as noted earlier. An alternative approach is the modification of acquisition parameters to select for a larger spatially encoded volume, thereby generating more signal per slice. At a gradient strength of 8.8 G cm^{-1}, shaped pulses adjusted for bandwidths of 6 kHz at frequencies offset by ± 21.5 kHz at a magnetic field strength of 9.4 T yield slices that are 1.7 mm thick. Reducing the spatial encoding gradient strength to 4.4 G/cm increases the slice thickness to 3.4 mm, thereby doubling the volume and the number of detected nuclei. However, to compensate for the effect this adjustment has on the position of the spatial encoding within the sample requires an offset adjustment of ± 10.75 kHz rather than ± 21.5 kHz (equivalent to setting cnst52/cnst53 to ± 30 ppm for measurements using a 9.4 T magnet). Care should be taken to ensure that Wavemaker generates the correct set of shaped pulses if such modifications are desired.

1.10 Automated measurement on Bruker AVANCE and AVANCE NEO systems under IconNMR.

To acquire data automatically under IconNMR for log*P* determination, the approach uses pulse schemes shown at **Figure S7c** and **S7d** alongside a series of macros and automation programs used for acquiring and post-acquisition processing the ^{19}F NMR data.

1.10.1 Instructions for setting up the NMR spectrometer.

^{2}H-locked and unlocked operation. For ^{2}H-capillary locked operation when a lock capillary is present, it’s recommended to create a lock table entry specifically for solvent selection when a capillary is present. In our practice, using Bruker Topspin command edlock to access the lock solvent table, the entry for solvent “D2O” was copied to a new line in the lock solvent table and renamed as “D2O_Cap”. This was then edited such that the loop gain value was altered from 5 to -22. This value was found to be suitable for stabilizing the NMR lineshape under slice-selective operations. Once installed, this solvent may be selected from the IconNMR solvent pull down menu. Within the IconNMR Configuration interface under Lock/Shim Options subsection Solvent/Probe Dependencies, the associated shim file entry for the “D2O_Cap” solvent should be left blank to prevent default shim file loading at the time of experiment execution. This allows for the shims to be set on a preceding sample

prior to insertion of a bilayer sample. Under the same Configuration window the associated shim routine should be set to "Skip Shimming (IconNMR not responsible)" in order to prevent shimming from occurring under automation.

For bilayer samples without a source of ^{2}H lock signal (unlocked data acquisition), we found it helpful to create a further new solvent entry within the lock solvent table that mirrored solvent "None" (no solvent). The new entry (e.g. named "None_FlogP") should be edited to disable it as a lock solvent (achieved by unticking the radio button against "Lock Solvent" within the Edit solvent parameters dialogue box. Thereafter, within the IconNMR Configuration interface, associated shim files for this solvent should be left blank, associated shim routine should be set to "Skip Shimming (IconNMR not responsible)" and associated lock routine should be set to "LOCK-OFF; #switch sweep and lock off". This "solvent" entry should be selected from the IconNMR experiment interface when a bilayer sample has no source of ^{2}H and will therefore run unlocked.

Parameter Source. For Bruker systems, two or three sets of parameters should be installed within the user parameter directory of the spectrometer.

The first parameter set, 19F_wsw, should be a copy of the standard 19F parameters where the sweep width, sw, has been set to a large value (e.g. 490 ppm) with the frequency offset adjusted to o1p = -100 ppm. A copy of this parameter set is available with the archive data and should be installed within the directory ~/exp/stan/nmr/par/user under the Topspin version directory of the relevant NMR spectrometer. Execution of paracon will enable the parameter set to be adapted for the spectrometer in question. This parameter set is called by the au program find19Fo1_run_FlogP and is used to run a scout scan to find the largest peak in the spectrum and set the offset, o1, for subsequent slice-selective data acquisitions.

The second parameter set, 19F_LogP (available from the data archive for both AVANCE and AVANCE NEO NMR spectrometers and adapted for the relevant NMR spectrometer by executing paracon to convert the parameter set for local use) calls the relevant pulse program and associated parameters for acquisition of ^{1}H-coupled ^{19}F NMR log*P* data. Once installed, the IconNMR Configuration should be edited to include the parameter set so that it may be called from the "Experiment" pull down menu within the IconNMR automation window. The parameter set includes the aunm entry "find19Fo1_run_FlogP" for automated data acquisition and the aunmp entry "lpp" for automated data processing. Further details of the operation of these processes and their associated code are presented below and are available from the data archive.

A third parameter set, 19F_Hdec_LogP, may also be installed in the same way provided the spectrometer hardware is configured for ^{1}H decoupling whilst observing ^{19}F. In this case, the relevant pulse program suitable for ^{19}F-{^{1}H} NMR spectroscopy is called. Installation and configuration should occur in the same way as described above for data acquisition without proton decoupling. Relevant parameter sets and all macros and au programs are available from the archive with program codes and explanations provided in the following section.

1.10.2 Code for pulse sequences and automation

For fully automated ^{19}F-based logP NMR data acquisition, program codes are provided under section "1.11 Code". These should be installed as follows:

a) Into the ~/exp/stan/nmr/au/src/user directory or equivalent of Topspin, add the automation programs "find19Fo1_run_FlogP", "au_wvm.selecho", "addp", "lpp" and "split2D_silent". "find19Fo1_run_FlogP" runs a scout scan to find the tallest ^{19}F NMR signal, sets the offset frequency, o1, to its frequency position, calls au_wvm.selecho to generate the relevant shaped pulses and executes the data acquisition according to the specified number of scans and relaxation delay. "split2D_silent" splits a pseudo 2D data set into separate 1D spectra. "addp" reads the data in procno 2, adds this to the data stored in procno 3 and writes the result (sum of spectra) into procno 4. The data in procno 4 (sum of spectra) can then be integrated manually from which the log*P* value is calculated.
b) Into the ~/exp/stan/nmr/lists/mac/user directory or equivalent of Topspin, add the macro "logpproc". This macro performs a Fourier transform of the acquired data along f2, phase corrects each row individually, baseline corrects along f2 and then calls au program "split2D_silent" followed by "addp".
c) Into the ~/exp/stan/nmr/lists/pp/user directory or equivalent of Topspin, add the pulse sequences "selgradgpigse2d" and "selgradgpse2d". These are provided as versions suitable for running under later versions of Topspin 3.x or under Topspin 4.x depending on spectrometer vintage.
d) Install the described parameter sets detailed under the previous section or create a parameter set for ^{19}F detection where the pulse program is either selgradgpse2d or selgradgpigse2d and set cnst52 and cnst53 to appropriate values to define the slice-selective pulse offset as described in the main article text and within this ESI. Ensure that aunm is set to find19Fo1_run_FlogP and that aunmp is set to lpp. For ease of implementation, suitable parameter sets and all programs are made available from

https://doi.org/10.15129/6ca1ffd8-270b-4ede-a3c0-91e083d57e21 together with example data set

1.11 Code

au program “addp”

```
/*********************************************************/
/*  addp      24.04.2024                                 */
/*********************************************************/
/*  Short Description :                                  */
/*  Calculate the sum of spectra from procnos.           */
/*********************************************************/
/*  Keywords :                                           */
/*  add, summation of spectra                            */
/*********************************************************/
/*  Description/Usage :                                  */
/*  Calculate the sum of spectra from procnos. The       */
/*  program assumes the current dataset is the one to be */
/*  added. It must be the first or last one of           */
/*  increasing procnos. The current data set is added    */
/*  in a loop to "num" subsequent procnos.               */
/*********************************************************/
/*  Adapted by JAP from previous substraction diffp au program   */
/*  Author(s) :                                          */
/*  Name:         Rainer Kerssebaum                      */
/*  Organisation:   Bruker BioSpin GmbH                  */
/*  Email:        rainer.kerssebaum@bruker-biospin.de    */
/*********************************************************/
/*  Name    Date    Modification:                        */
/*  rke     900827  created                              */
/*  rke     100716  bugfix for deleted ser-file          */
/*  rke     100716  bugfix for i1 not correctly initialised */
/*  jap     240424  modified to add two spectra together */
/*********************************************************/

int     first,add,num,tprocno;
double  swp;


  GETCURDATA
  add=procno+1;
  first=procno+2;
/*  GETINT("Enter first procno for addition : ",first)
  if (add==first)
    STOPMSG("program aborted\ncannot subtract from itself")
  if (first>add)
  { */ num=1;
/*    GETINT("Enter number of additions : ",num) */
    tprocno=(1+(int)((first+num)/10))*3+1;
/*  }
  else
  { num=add-first;
    tprocno=(1+(int)((add+1)/10))*3+1;
  }
  GETINT("Enter first target procno : ",tprocno);
*/
  DATASET(name,expno,first,disk,user)
```

```
  FETCHPARS("SW_p",&swp)

  for (i1=0;i1<num;i1++)
  { DATASET(name,expno,first+i1,disk,user)
    WRPPARAM(tprocno+i1)
    DATASET(name,expno,tprocno+i1,disk,user)
    STOREPARS("SW_p",swp)
    DATASET2(name,expno,first+i1,disk,user)
    DATASET3(name,expno,add,disk,user)
    STOREPAR("DC",1.0)

    ADD
  }
VIEWDATA_SAMEWIN
DATASET(name,expno,4,disk,user)
QUITMSG("--- sum of slices ---")

```

au program "find19Fo1_run_FlogP"

```
/****************************************************************/
/*       Find19Fo1 based on FindwaterTS3              22.08.2008        */
/****************************************************************/
/*       Short Description :                                            */
/*       AU program to automatically determine optimum o1p              */
/*       frequency centring 19F data acquisition on largest peak        */
/****************************************************************/
/*       Keywords :                                                     */
/*       19Fo1p                                                         */
/****************************************************************/
/*       Description/Usage :                                            */
/*       AU program using signal after 90 degree pulse                  */
/*    to optimise o1 frequency when carrying out FlogP protocols        */
/*       Does rga and zg, but limits rg to maximum of 128               */
/*    Run this from a dataset with pulprog set to the                   */
/*    sequence of your choice!                                          */
/****************************************************************/
/*       Author(s) :                                                    */
/*       Name            : Peter Gierth, Andrew Gibbs                   */
/*       Organisation    : Bruker UK                                    */
/*       Email           : peter.gierth@bruker.co.uk                    */
/*    Name               : John Parkinson - modified for FlogP          */
/*    Organization       : University of Strathclyde                    */
/****************************************************************/
/*       Name          Date                    Modification:            */
/*       ptg/agi    20080822                created                     */
/*       ptg        20100528 Updated for TS3 PROCPATH()syntax           */
/*                                 and power level parameters           */
/*  ptg             20150907     Sort out peak picking range to         */
/*                           be just around rough O1                    */
/*                           and set power based on RG                  */
/*  ptg    20150913       Add o1calib option (call as "findwaterTS3 o1calib")   */
/*  rss    20250612  use locnuc, remove back prediction of beginning of 19F     */
/*                 spectrum, remove extra RGA before wavemaker call     */
```

```
/*****************************************************************/

float PSH, PSP, IPS, maxpsh, maxpsp, maxips, cnst1;
char del[PATH_MAX], path[PATH_MAX], curp[PATH_MAX], locnuc[20];
double sf, sfo1, o1, sfo1real;
int noofscans, pscal_save;
FILE *fptr;
char pulprog[50];
int noofdummy;
int digmod;
char cmdsave[BUFSIZ];
char solvent[100];
char o1string[100];
int expnosave;
float p1, pl1;
int o1cal=0;
float rg, rffactor;
double f1p, f2p;
double bf;
double peakFreqHz, peakFreqPPM, peakIntensity;
int numPeaks, i;


GETCURDATA
expnosave = expno;

if (strcmp(cmd, "o1calib") == 0)
{
		o1cal =1 ;
}


FETCHPAR("P 1", &p1)
FETCHPAR("PLdB 1", &pl1)
FETCHPAR("LOCNUC", &locnuc);
FETCHPAR("O1", &o1)
FETCHPARS("RG", &rg)
expno=99998;
SETCURDATA

// setup 19F dataset

RPAR("19F_wsw", "all")
STOREPAR("PULPROG", "zg")
STOREPAR("O1", o1)
STOREPAR("P 1", p1)
STOREPAR("PLdB 1", pl1)
STOREPAR("NS",1)
STOREPAR("DS",0)
STOREPAR("RG",1.0)
STOREPAR("LOCNUC", locnuc)

ZG

FETCHPARS("BF1", &bf);
```

```
EF
MC
f1p=(o1/bf)+100.0;
f2p=(o1/bf)-100.0;

STOREPAR("F1P", f1p)
STOREPAR("F2P", f2p)
STOREPAR("PC", 2.0)
PP

/* find strongest peak */

numPeaks = readPeakList(PROCPATH(0));

   maxips=0.0;
   maxpsh=0.0;
   for (i=0; i<numPeaks; i++)
   {
          peakIntensity = getPeakIntensity(i);
          peakFreqHz = getPeakFreqHz(i);
          peakFreqPPM = getPeakFreqPPM(i);
          if (peakIntensity > maxips)
          {
             maxips = peakIntensity;
             maxpsh = peakFreqHz;
             maxpsp = peakFreqPPM;
          }
   }
   freePeakList();

FETCHPARS("SOLVENT", solvent)
FETCHPARS("BF1",&sf)
sfo1=sf + maxpsh * 1.0e-6;

expno=expnosave;
SETCURDATA

// store correct o1

STOREPAR("SFO1",sfo1);
XAU("au_wvm.selecho","")
QUIT
```

au program “lpp”

```
/**********************************************************/
/* au program for aunmp entry to call logpproc for processing FlogP data */
/* JAP University of Strathclyde 04/2025                              */
/*********************************************************/
XMAC("logpproc")
QUIT
```

au program “split2d_silent”

```
/*******************************************************************/
/*      split2D_silent                    24.04.2024               */
/*******************************************************************/
/*      Short Description :                                        */
/*      Program which splits a processed 2D file into single       */
/*      1D spectra, extracting 2 slices only without prompting     */
/*******************************************************************/
/*******************************************************************/
/*      Description/Usage :                                        */
/*      Program which splits a logP processed 2D file into two     */
/*      single 1D spectra.                                         */
/*******************************************************************/
/*  Based on original author work by Rainer Kerssebaum             */
/*      Author(s) :                                                */
/*      Name            : Rainer Kerssebaum                        */
/*      Organisation    : Bruker Analytik                          */
/*      Email           : rainer.kerssebaum@bruker.de              */
/*  Modified John A. Parkinson                                     */
/*  Organisation  : University of Strathclyde                      */
/*******************************************************************/
/*      Name            Date    Modification:                      */
/*      rke             910827 created                             */
/*      jap             240424 modified                            */
/*******************************************************************/

int si, tprocno;

GETCURDATA
FETCHPAR1S("SI",&si)

for (i1=1;i1<=si;i1++)
   RSR(i1,i1+tprocno-1)
IPROCNO
VIEWDATA_SAMEWIN
DATASET(name,expno,2,disk,user)
QUIT
```

au program “au_wvm.selecho”

```
/*******************************************************************/
/*      au_wvm.selecho based on au_wvm from 12.12.2019             */
/*******************************************************************/
/*      Short Description :                                        */
/*      General AU program for data acquisition.                   */
/*******************************************************************/
/*      Keywords :                                                 */
/*      zg                                                         */
/*******************************************************************/
/*      Description/Usage :                                        */
/*      General AU program for data acquisition.                   */
/*      Create shaped pulses as defined in the pulse program using WaveMaker */
/*******************************************************************/
```

```
/*        Author(s) :                                                         */
/*        Name              : Peter Kiraly                                    */
/*        Organisation      : University of Manchester                        */
/*        Email             : peter.kiraly@manchester.ac.uk                   */
/****************************************************************************/
/*        Name              Date      Modification:                           */
/*        pdv               191212 created                                    */
/*        rss               250221 modified for use with pp selgradgpse2d.rss */
/*                                                                            */
/****************************************************************************/

double o1p, offset;
offset = 230.0;

GETCURDATA
FETCHPAR("O1P", &o1p)
STOREPAR("CNST 52", o1p+offset)
STOREPAR("CNST 53", o1p-offset)
XCMD("wvm -q")
XAU("au_zg","")
QUIT
```

macro “logpproc”

```
# Procedure for automated processing of 19FlogP NMR data
#
# Step 1 - Performs f2 transformation on current pseudo 2D
# Step 2 - Phase corrects each row individually
# Step 3 - Baseline corrects in f2
# Step 4 - Extract 2 spectra then write them to procno 2 and procno 3 using au program split2D_silent
# Step 5 - Read procno 2, add procno 3
# Step 6 - Write to procno 4 and display result
xf2
diff_apk2d 1
abs2
split2D_silent
addp
```

Pulse Programs for AVANCE NEO systems running under Topspin version 4.x

"selgradgpse2d" for ^{19}F NMR data acquisition with proton coupling

```
;Based on selgpse
;avance-version (21/09/15)
;1D spin echo with gradients
;   using selective refocussing with a shaped pulse
;   in the presence of a gradient for slice selectivity
;   pseudo-2D for selecting two slices
;   uses wavemaker to create the two pulses
;
;$CLASS=HighRes
;$DIM=1D
;$TYPE=
;$SUBTYPE=
;$COMMENT=


#include <Avance.incl>
#include <Delay.incl>
#include <Grad.incl>

create_shape(sp2, rsnob, name= ref_plus60, stepSize=10.0 us, bandwidth=6000 Hz, offset=$cnst52
ppm)
create_shape(sp3, rsnob, name= ref_minus60, stepSize=10.0 us, bandwidth=6000 Hz, offset=$cnst53
ppm)
"TAU1=de"
"TAU2=p1*2/PI"


#   ifdef CALC_SPOFFS
"spoffs2=bf1*(cnst21/1000000)-o1"
#   else
#   endif /*CALC_SPOFFS*/

"l0=1"

"acqt0=0"
baseopt_echo

1 ze
2 30m

#   ifdef FLAG_BLK
  4u BLKGRAD
#   else
  4u
#   endif /*FLAG_BLK*/

  d1 pl1:f1
  50u UNBLKGRAD

  p1 ph1
```

```
 TAU1
 p16:gp2
 d16
 5u gron1
 if "l0 %2 == 1"
 {
  p12:sp2:f1 ph2:r
 }
 else
 {
  p12:sp3:f1 ph2:r
 }
 5u groff
 p16:gp2
 d16

#   ifdef FLAG_BLK
 TAU2
#   else
 TAU2 BLKGRAD
#   endif /*FLAG_BLK*/

 go=2 ph31
 30m mc #0 to 2 F1QF(calclc(l0,1))

#   ifdef FLAG_BLK
 4u BLKGRAD
#   else
 4u
#   endif /*FLAG_BLK*/
exit

ph1=0 0 3 3 2 2 1 1
ph2=1 3
ph31=0 0 1 1 2 2 3 3

;pl1 : f1 channel - power level for pulse (default)
;sp2: f1 channel - shaped pulse – first offset frequency
;sp3: f1 channel – shaped pulse – second offset frequency
;p1 : f1 channel -  90 degree high power pulse
;p12: f1 channel - 180 degree shaped pulse
;p16: homospoil/gradient pulse                         [1 msec]
;d1 : relaxation delay; 1-5 * T1
;d16: delay for homospoil/gradient recovery
;cnst21: chemical shift for selective pulse (offset, in ppm)
;ns: 2 * n, total number of scans: NS * TD0
;ds: 4
;phcor 2 : phasedifference between power levels sp1 and pl1
;choose p12 according to desired selectivity
;the flip-angle is determined by the amplitude
;set O1 on resonance on the multiplet to be excited or use spoffs

;use gradient ratio:    gp 16
;                       45
```

```
;for z-only gradients:
;gpz1: 16% (for selectivity)
;gpz2: 45%

;use gradient files:
;gpnam1: SMSQ10.100

;preprocessor-flags-start
;CALC_SPOFFS: automatically calculate spoffs for selective pulses
;            start experiment with option -DCALC_SPOFFS (eda: ZGOPTNS)
;FLAG_BLK: for BLKGRAD before d1 rather than go
;option -DFLAG_BLK: (eda: ZGOPTNS)
;preprocessor-flags-end

;$Id: $
```

"selgradgpigse2d" for ^{19}F-{^{1}H} NMR data acquisition with proton decoupling

```
;Based on selgpse
;avance-version (21/09/15)
;1D spin echo with gradients
;   using selective refocussing with a shaped pulse
;   in the presence of a gradient for slice selectivity
;   pseudo-2D for selecting two slices
;   use wavemaker to create the two pulses
;   with decoupling
;   RSS 2025/2/23
;
;$CLASS=HighRes
;$DIM=1D
;$TYPE=
;$SUBTYPE=
;$COMMENT=

#include <Avance.incl>
#include <Delay.incl>
#include <Grad.incl>

create_shape(sp2, rsnob, name= ref_plus60, stepSize=10.0 us, bandwidth=6000 Hz, offset=$cnst52
ppm)
create_shape(sp3, rsnob, name= ref_minus60, stepSize=10.0 us, bandwidth=6000 Hz, offset=$cnst53
ppm)
"TAU1=de"
"TAU2=p1*2/PI"

#   ifdef CALC_SPOFFS
"spoffs2=bf1*(cnst21/1000000)-o1"
#   else
#   endif /*CALC_SPOFFS*/

"l0=1"
"d11=30m"
```

```
"acqt0=0"
baseopt_echo
1 ze
 d11 pl12:f2
2 30m do:f2
#   ifdef FLAG_BLK
 4u BLKGRAD
#   else
 4u
#   endif /*FLAG_BLK*/
 d1 pl1:f1
 50u UNBLKGRAD
 p1 ph1
 TAU1
 p16:gp2
 d16
 5u gron1
 if "l0 %2 == 1"
 {
  p12:sp2:f1 ph2:r
 }
 else
 {
  p12:sp3:f1 ph2:r
 }
 5u groff
 p16:gp2
 d16
#   ifdef FLAG_BLK
 TAU2
#   else
 TAU2 BLKGRAD
#   endif /*FLAG_BLK*/
 go=2 ph31 cpd2:f2
 30m do:f2 mc #0 to 2 F1QF(calclc(l0,1))
#   ifdef FLAG_BLK
 4u BLKGRAD
#   else
 4u
#   endif /*FLAG_BLK*/
exit
ph1=0 0 3 3 2 2 1 1
ph2=1 3
ph31=0 0 1 1 2 2 3 3
```

```
;pl1 : f1 channel - power level for pulse (default)
;pl12: f2 channel - power level for CPD/BB decoupling
;sp2: f1 channel - shaped pulse for first frequency offset
;sp3: f1 channel – shaped pulse for second frequency offset
;p1 : f1 channel -  90 degree high power pulse
;p12: f1 channel - 180 degree shaped pulse
;p16: homospoil/gradient pulse                          [1 msec]
;d1 : relaxation delay; 1-5 * T1
;d16: delay for homospoil/gradient recovery
;cnst21: chemical shift for selective pulse (offset, in ppm)
;ns: 2 * n, total number of scans: NS * TD0
;ds: 4
;cpd2: decoupling according to sequence defined by cpdprg2
;pcpd2: f2 channel - 90 degree pulse for decoupling sequence

;phcor 2 : phasedifference between power levels sp1 and pl1

;choose p12 according to desired selectivity
;the flip-angle is determined by the amplitude
;set O1 on resonance on the multiplet to be excited or use spoffs

;use gradient ratio:    gp 16
;                          15

;for z-only gradients:
;gpz1: 16% (for selectivity)
;gpz2: 45%

;use gradient files:
;gpnam1: SMSQ10.100

;preprocessor-flags-start
;CALC_SPOFFS: automatically calcuate spoffs for selective pulses
;start experiment with option -DCALC_SPOFFS (eda: ZGOPTNS)
;FLAG_BLK: for BLKGRAD before d1 rather than go
;option -DFLAG_BLK: (eda: ZGOPTNS)
;preprocessor-flags-end

;$Id: $
```

Pulse Programs for AVANCE systems running under Topspin version 3.x

“selgradgpse2d.ts3” for ^{19}F NMR data acquisition with proton coupling

```
;Based on selgpse
;avance-version (21/09/15)
;1D spin echo with gradients
;   using selective refocussing with a shaped pulse
;   in the presence of a gradient for slice selectivity
;   pseudo-2D for selecting two slices
;   use wavemaker to create the two pulses
```

```
;
;RSS 2025/2/23
;uses wvm syntax rather than create_shape for compatibility with TS 3.7
;
;$CLASS=HighRes
;$DIM=1D
;$TYPE=
;$SUBTYPE=
;$COMMENT=


#include <Avance.incl>
#include <Delay.incl>
#include <Grad.incl>

"TAU1=de"
"TAU2=p1*2/PI"


#   ifdef CALC_SPOFFS
"spoffs2=bf1*(cnst21/1000000)-o1"
#   else
#   endif /*CALC_SPOFFS*/

"l0=1"

"acqt0=0"
baseopt_echo


1 ze
2 30m

#   ifdef FLAG_BLK
  4u BLKGRAD
#   else
  4u
#   endif /*FLAG_BLK*/

  d1 pl1:f1
  50u UNBLKGRAD

  p1 ph1
  TAU1
  p16:gp2
  d16
  5u gron1
  if "l0 %2 == 1"
  {
    p12:sp2:f1 ph2:r
  }
  else
  {
    p12:sp3:f1 ph2:r
  }
```

```
 5u groff
 p16:gp2
 d16

# ifdef FLAG_BLK
 TAU2
# else
 TAU2 BLKGRAD
# endif /*FLAG_BLK*/

 go=2 ph31
 30m mc #0 to 2 F1QF(calclc(l0,1))

# ifdef FLAG_BLK
 4u BLKGRAD
# else
 4u
# endif /*FLAG_BLK*/

exit

ph1=0 0 3 3 2 2 1 1
ph2=1 3
ph31=0 0 1 1 2 2 3 3

;pl1 : f1 channel - power level for pulse (default)
;sp2: f1 channel - shaped pulse for first frequency offset
;sp3: f1 channel – shaped pulse for second frequency offset
;p1 : f1 channel -  90 degree high power pulse
;p12: f1 channel - 180 degree shaped pulse
;p16: homospoil/gradient pulse                    [1 msec]
;d1 : relaxation delay; 1-5 * T1
;d16: delay for homospoil/gradient recovery
;cnst21: chemical shift for selective pulse (offset, in ppm)
;ns: 2 * n, total number of scans: NS * TD0
;ds: 4

;phcor 2 : phasedifference between power levels sp1 and pl1

;choose p12 according to desired selectivity
;the flip-angle is determined by the amplitude
;set O1 on resonance on the multiplet to be excited or use spoffs

;use gradient ratio:    gp 15
;                          45

;for z-only gradients:
;gpz1: 16% (for selectivity)
;gpz2: 45%

;use gradient files:
;gpnam1: SMSQ10.100
```

```
;preprocessor-flags-start
;CALC_SPOFFS: automatically calcuate spoffs for selective pulses
;start experiment with option -DCALC_SPOFFS (eda: ZGOPTNS)
;FLAG_BLK: for BLKGRAD before d1 rather than go
;option -DFLAG_BLK: (eda: ZGOPTNS)
;preprocessor-flags-end

;WaveMaker shapes (optional)
;use 'wvm -a' command to create the necessary shape files
;sp2:wvm: rsnob(6000 Hz, cnst52 ppm; ss=10 us)
;sp3:wvm: rsnob(6000 Hz, cnst53 ppm; ss=10 us)
;cnst52: offset for pulse at +60 defined by au_wvm.selecho and hard coded
;cnst53: offset for pulse at -60 defined by au_wvm.selecho and hard coded

;$Id: $
```

"selgradgpigse2d.ts3" for ^{19}F-{^{1}H} NMR data acquisition with proton decoupling

```
;Based on selgpse
;avance-version (21/09/15)
;1D spin echo with gradients
;   using selective refocussing with a shaped pulse
;   in the presence of a gradient for slice selectivity
;   pseudo-2D for selecting two slices
;   use wavemaker to create the two pulses
;with decoupling
;RSS 2025/2/23
;uses wvm syntax rather than create_shape for compatibility with TS 3.7
;
;$CLASS=HighRes
;$DIM=1D
;$TYPE=
;$SUBTYPE=
;$COMMENT=

#include <Avance.incl>
#include <Delay.incl>
#include <Grad.incl>

"TAU1=de"
"TAU2=p1*2/PI"

#   ifdef CALC_SPOFFS
"spoffs2=bf1*(cnst21/1000000)-o1"
#   else
#   endif /*CALC_SPOFFS*/

"l0=1"
"d11=30m"

"acqt0=0"
```

```
baseopt_echo

1 ze
  d11 pl12:f2
2 30m do:f2

#   ifdef FLAG_BLK
  4u BLKGRAD
#   else
  4u
#   endif /*FLAG_BLK*/

  d1 pl1:f1
  50u UNBLKGRAD

  p1 ph1
  TAU1
  p16:gp2
  d16
  5u gron1
  if "l0 %2 == 1"
  {
    p12:sp2:f1 ph2:r
  }
  else
  {
    p12:sp3:f1 ph2:r
  }
  5u groff
  p16:gp2
  d16

#   ifdef FLAG_BLK
  TAU2
#   else
  TAU2 BLKGRAD
#   endif /*FLAG_BLK*/

  go=2 ph31 cpd2:f2
  30m do:f2 mc #0 to 2 F1QF(calclc(l0,1))

#   ifdef FLAG_BLK
  4u BLKGRAD
#   else
  4u
#   endif /*FLAG_BLK*/

exit


ph1=0 0 3 3 2 2 1 1
```

```
ph2=1 3
ph31=0 0 1 1 2 2 3 3


;pl1 : f1 channel - power level for pulse (default)
;pl12: f2 channel - power level for CPD/BB decoupling
;sp2: f1 channel - shaped pulse for first offset frequency
;sp3: f1 channel – shaped pulse for second offset frequency
;p1 : f1 channel -  90 degree high power pulse
;p12: f1 channel - 180 degree shaped pulse
;p16: homospoil/gradient pulse                    [1 msec]
;d1 : relaxation delay; 1-5 * T1
;d16: delay for homospoil/gradient recovery
;cnst21: chemical shift for selective pulse (offset, in ppm)
;ns: 2 * n, total number of scans: NS * TD0
;ds: 4
;cpd2: decoupling according to sequence defined by cpdprg2
;pcpd2: f2 channel - 90 degree pulse for decoupling sequence

;phcor 2 : phasedifference between power levels sp1 and pl1

;choose p12 according to desired selectivity
;the flip-angle is determined by the amplitude
;set O1 on resonance on the multiplet to be excited or use spoffs

;use gradient ratio:    gp 16
;                       45

;for z-only gradients:
;gpz1: 16% (for selectivity)
;gpz2: 45%

;use gradient files:
;gpnam1: SMSQ10.100

;preprocessor-flags-start
;CALC_SPOFFS: automatically calcuate spoffs for selective pulses
;start experiment with option -DCALC_SPOFFS (eda: ZGOPTNS)
;FLAG_BLK: for BLKGRAD before d1 rather than go
;option -DFLAG_BLK: (eda: ZGOPTNS)
;preprocessor-flags-end

;WaveMaker shapes (optional)
;use 'wvm -a' command to create the necessary shape files
;sp2:wvm: rsnob(6000 Hz, cnst52 ppm; ss=10 us)
;sp3:wvm: rsnob(6000 Hz, cnst53 ppm; ss=10 us)
;cnst52: offset for pulse at +60 defined by au_wvm.selecho and hard coded
;cnst53: offset for pulse at -60 define by au_wvm.selecho and hard coded

;$Id: $
```

2.0 References

1. S. Purser, P. R. Moore, S. Swallow and V. Gouverneur, *Chem. Soc. Rev.*, doi = 10.1039/B610213C, **37**(2), 320-330
2. B. Linclau, Z. Wang, G. Compain, V. Paumelle, C. Q., Angew. Chem. Int. Ed., 2016, doi = 10.1002/anie.201509460, 55, 674-678.
3. T. Fujita, J. Iwasa and C. Hansch, *J. Am. Chem. Soc.,* 1964, **86**(23), 5175-5180.
4. D. E. Leahy, J. J. Morris, P. J. Taylor and A. R. Wait, *J. Chem. Soc. Perkin Trans. 2,* 1992, **4**, 723-731.
5. C. M. Ellison, M. T.D Cronin, J. C. Madden and T. W. Schultz, *SAR and QSAR in Environmental Research*, 2008, **7-8**, 751-783.
6. Y. Ben-Tal and J. E. Hein, *J. Org. Chem.,* 2025, doi = 10.1021/acs.joc.5c00717, 90, 23, 7832–7840.
7. Y. N. Mitrev, *Bulg. Chem. Comm.* 2017, **49**(Special Issue F), 64-69.
